# Estradiol, Emotion Regulation, and the Limbic System: Effects on Gray Matter Volume

**DOI:** 10.1016/j.bpsgos.2026.100709

**Published:** 2026-02-17

**Authors:** Anna Franziska Denninger, Elisa Rehbein, Inger Sundström-Poromaa, Birgit Derntl, Lydia Kogler

**Affiliations:** a Department of Psychiatry and Psychotherapy, Tübingen Center for Mental Health, University of Tübingen, Tübingen, Germany; b Department of Psychiatry and Psychotherapy, Philipps-Universität Marburg, Marburg, Germany; c Department of Women’s and Children’s Health, Uppsala University, Uppsala, Sweden; d German Centre for Mental Health, partner site Tübingen, Germany; e LEAD Graduate School, University of Tübingen, Tübingen, Germany

**Keywords:** Emotion regulation, Estradiol, Gonadal steroid hormones, Menstrual cycle, Neostriatum, Randomized controlled trial

## Abstract

**Background:**

Mastering emotion regulation is crucial for social skills and mental health. Hormonal fluctuations, particularly in estradiol (E2) levels across the menstrual cycle, significantly impact emotion processing. E2 is known to influence emotion regulation, mental health, and the plasticity of limbic and striatal regions, which are involved in emotion processing and are rich in E2 receptors. Although research indicates that E2 levels may impact gray matter volume (GMV) of limbic and striatal areas, sufficient causal evidence is missing so far. Furthermore, because of the additional fluctuations of progesterone across the menstrual cycle, the sole impact of E2 on brain volume has been difficult to disentangle.

**Methods:**

To isolate the effects of E2 from other fluctuating sex hormones, we used a randomized, placebo-controlled, double-blind, crossover design and administered oral E2 to 27 naturally cycling females during their early follicular phase (low endogenous sex hormone levels). We analyzed emotion regulation strategies and E2 levels to assess their impact on regional GMV.

**Results:**

Our data showed that a rapid increase of E2 was negatively associated with right striatal GMV. Moreover, greater use of reappraisal was associated with reduced GMV of the right striatum. Rapidly increased E2 did not influence GMV of other limbic regions.

**Conclusions:**

These results highlight that rapid increases in E2 and individual differences in emotion regulation dynamically modulate GMV. This offers important implications for female mental and brain health associated with hormonal fluctuations. Considering female endocrine profiles improves hormone-informed health care and therefore supports individualized medicine.

## Plain Language Summary

This study looks at how changes in the hormone estradiol (E2) and emotion regulation strategies affect brain structure in naturally cycling females. To isolate E2’s effect, E2 levels were pharmacologically increased in 27 females at a time in their menstrual cycle when natural hormone levels are low. We found that a rapid rise in E2 was linked to reduced volume in the right striatum, a brain area involved in emotion processing. How people regulate emotions also related to these brain changes, highlighting the importance of hormone-aware mental health care.

Estradiol (E2) is traditionally viewed as a reproductive hormone, synthesized primarily in the ovaries and, to a lesser extent, in the adipose tissue of females (1). Importantly, E2 is also synthesized de novo within the brain (2), where peripheral and neuro-derived E2 can act locally via estrogen receptors (ERs) to exert neuromodulatory (3,4) and potentially neuroprotective effects (5), driving morphological and neurochemical changes. E2-induced effects in the brain include modulation of synaptic plasticity, regulation of neurotransmitter systems, reduction of oxidative stress and inflammation, promotion of neuronal growth, plasticity via regulation of BDNF (brain-derived neurotrophic factor), cell signaling pathways, and transcription factor activation (5).

However, E2 levels constantly change throughout the female reproductive lifespan. During the menstrual cycle, E2 levels are low during the early follicular phase, peak shortly before ovulation, and drop and then peak again together with progesterone (P4) during the luteal phase before both hormones rapidly decline just before menstruation (6). Across the menstrual cycle, high-E2 phases (such as the preovulatory phase) are linked to increased gray matter volume (GMV) in the hippocampus (7,8) and anterior cingulate cortex (ACC) (9) but decreased GMV in the striatum (9,10) compared with low-E2 phases (such as the early follicular or late luteal phase). However, findings for the amygdala are mixed (11, 12, 13). These findings suggest that the hippocampus, ACC, amygdala, and striatum exhibit structural plasticity in response to endogenous fluctuating E2 levels, with higher GMV of the hippocampus and ACC and lower GMV of the striatum when E2 levels are high vs. low. These areas are central to affective processing and the regulation of behavioral and physiological responses to emotional stimuli. Importantly, these regions are also rich in ERs, indicating that they may be sensitive to changes in E2 levels (14,15). This E2 fluctuation–specific responsivity may involve changes in synaptic structure and function (2) and therefore may be associated with affect and cognition (2,16).

Endogenous E2 fluctuations as well as phases of altered E2 exposure modulate neuronal emotion regulation, emotion processing, and functional connectivity of the emotion regulation network (4,17,18). E2 administration has been shown to alter activity patterns during reappraisal use (4) and change effective network connectivity (17). Individuals can regulate their emotions by applying a variety of strategies, and the effectiveness of emotion regulation strategies varies between menstrual cycle phases. Reappraisal, a cognitive emotion regulation strategy, is based on changing the meaning of situations to alter emotional responsiveness (19,20). Reappraisal successfully reduces negative affect (21), and higher E2 levels are associated with more effective reappraisal use (22). Consequently, when E2 levels are low (as in the early follicular phase), individuals show increased effort to apply reappraisal but with reduced success (21). Rumination is a regulation strategy with a repetitive and recurrent focus on negative thoughts or emotions (23). It is often associated with increased anxiety and stress levels (24,25) and poor mental health (26,27). Research has shown that females with low E2 who engage in rumination experience greater negative affect (26). Some evidence suggests that emotion regulation traits such as reappraisal influence feelings of sadness differently throughout the menstrual cycle (28). Similarly, Rafiee *et al.* (29) demonstrated a negative association between emotion regulation traits and emotion recognition when P4 levels were elevated. These findings suggest that individual differences in emotion regulation abilities may shape emotional experiences in the context of hormonal changes.

Furthermore, GMV is related to trait use of emotion regulation strategies. Reappraisal has been negatively associated with ventral striatum GMV (30) and positively associated with amygdala GMV (31). Regarding rumination, positive associations have been reported with GMV in the parahippocampus (32) and the caudate nucleus (part of the striatum) (33), while negative associations have emerged with the left ACC GMV (34). It should be noted that the proportion of female participants varied in these studies, as did their reproductive and hormonal status (from adolescents to postmenopausal women), which might have influenced the observed associations between emotion regulation traits and GMV.

Although an association between E2 levels and the application of emotion regulation strategies is assumed, the literature is scarce regarding how E2 and different emotion regulation traits affect GMV in emotion processing regions and whether these 2 variables interact in their influence on GMV of these regions. Therefore, in the current study, we aimed to investigate the effect of E2 and emotion regulation traits on GMV of regions involved in emotion processing. By experimentally increasing E2 levels in a randomized, placebo-controlled, crossover design during the early follicular phase, we were able to compare elevated E2 levels with naturally low E2 levels and investigate the variability in E2 levels, which appear to be the driving factor for structural changes rather than absolute levels (13). Additionally, the assessment of reappraisal and rumination trait use gives us insight into the impact of E2 levels on brain plasticity in females applying reappraisal or rumination. With this design, we wanted to examine whether 1) increasing E2 levels are positively associated with greater GMV in the hippocampus and ACC (7,35) and negatively associated with GMV in the amygdala and striatum (9,10,12) and 2) whether the use of cognitive emotion regulation strategies such as reappraisal and rumination differentially affects brain structure. Given the limited and inconclusive literature, we hypothesized on an exploratory basis that amygdala, hippocampus, ACC, and striatum GMVs would be associated with emotion regulation strategies. Specifically, we hypothesized that trait use of cognitive reappraisal would be positively associated with amygdala volume (31) and negatively associated with smaller striatum volumes (30). Rumination is associated with larger striatum (33) and smaller ACC GMV (34). Additionally, we hypothesized that the trait use of emotion regulation strategies would interact with E2 levels influencing brain volume. For cognitive reappraisal, we assumed that higher trait use would be associated with smaller GMV of the striatum when E2 levels are high. For the other regions, no direct hypothesis can be formulated.

## Methods and Materials

### Sample Description

Inclusion criteria were female sex assigned at birth, ages 19 to 35 years, right handedness, and a regular menstrual cycle (26–32 days). To ensure the regularity of the menstrual cycle, 2 cycles were tracked before participation (report of menstruation onset). Participants were excluded if they had any magnetic resonance imaging (MRI) contraindication (e.g., metal implants); present or past mental (Structured Clinical Interview for DSM) (36), neurological, or endocrine disorders; use of hormonal contraceptives during the last 6 months; medication intake; and past or present pregnancies. All participants signed an informed consent form. The study was approved by the Ethics Committee of the Medical Faculty, University of Tübingen (754/2017/BO1) and preregistered on ClinicalTrials.gov (NCT06312033), and data were collected in Tübingen, Germany, between 2018 and 2019.

### Procedure

**Figure 1:**
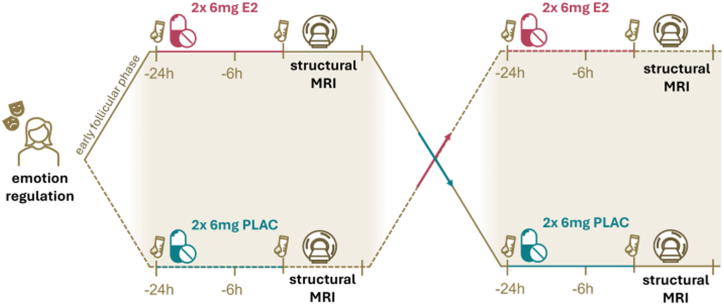
Study procedure. Assessment of trait use of emotion regulation including reappraisal and rumination followed by 2 consecutive drug administrations (estradiol [E2] or placebo [PLAC]) 24 hours and 6 hours before a structural magnetic resonance imaging (MRI) scan. Before drug administration and after MRI measurement, blood samples were retrieved to assess hormone levels. This procedure was repeated after a minimum of 2 months with the other drug condition.

We applied a randomized, placebo-controlled, double-blind, crossover design for the E2 administration. After an initial screening and a singular assessment of variables for sample description and emotion regulation (Heidelberg Form for Emotion Regulation Strategies) (37), participants reported the onset of their menstruation and were invited to the study within 2 to 5 days after menstruation onset to capture the early follicular phase. To safely and efficiently increase E2 levels comparable with preovulatory levels (3,38), E2-valerate (6 mg, Progynova21; Bayer Weimar GmbH & Co. KG) or placebo pills (PLAC) were administered approximately 24 hours and 6 hours before the MRI assessment in a randomized double-blind manner. Because peak E2 concentrations are typically reached within 4 to 9 hours, and repeated dosing approximately doubles E2 levels, we applied sequential dosing to ensure a stable E2 peak (product information of Progynova21; Bayer Weimar GmbH & Co. KG). Before the MRI assessment, depression symptoms (Beck Depression Inventory-II [BDI-II]) (39) and state and trait anxiety (State-Trait Anxiety Inventory [STAI]) (40) were assessed. After a washout period of at least 2 months, the participants crossed over to the other drug condition (E2/PLAC), and the procedures were repeated (Figure 1). As this study was part of a larger study, also see Rehbein *et al.* (4,41) and Derntl *et al.* (17) for more detailed descriptions of the study procedure.

### Hormone Assessment

Blood samples of hormone levels (E2, P4, testosterone) were collected before E2/PLAC intake and after the MRI session by the central laboratory, University Hospital Tübingen, and analyzed using chemiluminescence immunoassay. Sensitivity ranges were as follows: E2 = 43.6–11,010 pmol/L; P4 = 0.67–190.8 nmol/L; testosterone = 0.24–52.05 nmol/L.

### Neuroimaging

Structural MRI data were acquired by a Siemens Prisma 3T scanner (University Hospital Tübingen). T1-weighted images were obtained with a standard magnetization-prepared rapid acquisition gradient-echo (MPRAGE) sequence (TR = 2300 ms, TE = 4.16 ms, slice thickness = 1.00 mm, voxel size = 1 × 1 × 1 mm, flip angle of 9°, distancing factor 50%, GRAPPA acceleration factor, sagittal orientation) and a 64-channel head coil.

Data were preprocessed using the CAT12 toolbox (https://neuro-jena.github.io/cat/), SPM12 (https://www.fil.ion.ucl.ac.uk/spm/software/spm12), and MATLAB (version R2024; The MathWorks, Inc.). T1-weighted images underwent normalization; longitudinal segmentation into cerebrospinal fluid, white matter volume, and GMV; warping to Montreal Neurological Institute (MNI) space, and smoothing with a full width at half maximum isotropic Gaussian kernel of 8 mm. Total intracranial volume (TIV) and GMV were extracted.

**Figure 2:**
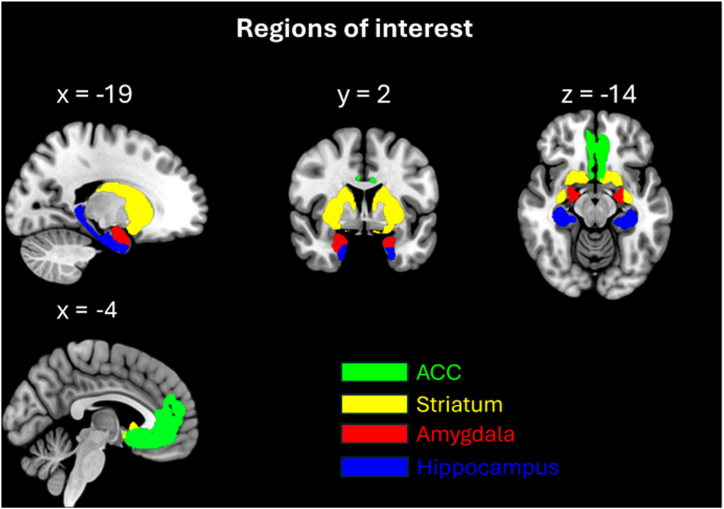
Regions of interest in the Montreal Neurological Institute space: anterior cingulate cortex (ACC) (green), striatum (yellow), amygdala (red), and hippocampus (blue). Additional slices are provided in Figure S1.

Regions of interest (ROIs) (Figure 2) included the bilateral hippocampus, ACC, amygdala, and striatum. To inspire future studies, we also exploratorily investigated subregions (dorsal, ventral, and caudoventral striatum; [para]hippocampus; dorsal, pre, and subgenual ACC), which are reported in the Supplement. ROI masks were defined according to the JUBrain toolbox (42), except for the striatum, which was defined according to Liu *et al.* (43). Masks were further extracted with an adapted get_totals script (http://www0.cs.ucl.ac.uk/staff/gridgway/vbm/get_totals.m) as was done previously (10).

### Statistical Analysis

Statistical analyses were performed in MATLAB. Differences between drug conditions in behavioral, hormonal, and structural data were examined using dependent *t* tests or Wilcoxon signed-rank tests when assumptions for parametric testing were not met. Nonparametric effect size was calculated using Rosenthal’s *r*, where $r=\frac{z}{\sqrt{n}\;}$, following Rosenthal (44).

To assess the effect of 1) E2 increase in the E2 or PLAC condition (E2 levels pre minus postdrug administration [E2Δ]) and 2) emotion regulation trait (reappraisal, rumination) on GMV, individual robust multiple linear regression models were applied with MATLAB’s built-in robust model option (less sensitive to outliers). TIV and age were applied as covariates, resulting in

$$1.\text{GMV}\;∼\;{E2\Delta }_{E2\;|\;\text{PLAC}}\;+\;\text{age}\;+\;\text{TIV}$$

$$2.\text{GMV}\;∼\;{\text{trait}}_{\text{reappraisal}\;|\;\text{rumination}}+\text{age}\;+\;\text{TIV, and}$$

(1) $$3.\text{GMV}\;∼\;{E2\Delta }_{E2\;|\;\text{PLAC}}\times {\text{trait}}_{\text{reappraisal}\;|\;\text{rumination}}\;+\;\text{age}\;+\;\text{TIV.}$$

E2 increase was expressed as positive values and log transformed. All predictors and covariates were centered relative to their mean. Multiple linear regression was selected based on the reduced risk of overfitting given our small sample size and the fact that we analyzed the 2 drug conditions separately. To evaluate differences between the drug conditions, beta values were calculated, *z* transformed, and then tested for significance applying the cumulative distribution function of the standard normal distribution.

(2) $$z=\frac{\beta 1-\beta 2}{\sqrt{{SE}_{1}^{2}+{SE}_{2}^{2}}}$$

The level of significance was set at *p* = .05 and corrected for multiple comparisons (2 hemispheres, 4 ROIs) by applying false discovery rate (FDR) correction using the Benjamini-Hochberg (45) procedure. Sample size was determined via G-power analysis reported by Rehbein *et al.* (4).

## Results

### Sample Description

Initially, 32 naturally cycling females participated in the study. Five participants had to be excluded due to missing MRI data (*n* = 1), start of hormonal contraception use between the 2 sessions (*n* = 1), and depressive symptoms at one of the 2 drug condition sessions (BDI-II scores ≥20; *n* = 3). Thus, the final sample size included 27 females with a mean age of 23.22 years (Table 1). State anxiety (*t*_26_ = −0.042, *p* = .97) and BDI-II scores (*t*_26_ = −0.189, *p* = .85) prior to drug administration did not differ between drug conditions, and effects did not depend on the order of drug administration (Table 1).

**Table 1:** Sample Description

	Mean ± SD	Estradiol	Placebo	*p* Value
Age, Years	23.22 ± 3.0	–	–	–
Verbal Intelligence	32.59 ± 3.2	–	–	–
Reappraisal Trait	15.15 ± 2.7	–	–	–
Rumination Trait	14.07 ± 3.5	–	–	–
Anxiety, Trait	33.59 ± 7.4	–	–	–
Anxiety, State	–	38.04 ± 6.4	38.11 ± 8.1	.967
Depression Score	–	6.26 ± 4.9	6.33 ± 5.3	.852

Values are presented as mean ± SD. The reappraisal trait and the rumination trait were measured with the Heidelberg Form for Emotion Regulation Strategies and anxiety was measured with the State-Trait Anxiety Inventory.

### Effective Elevation of E2 Levels

E2 levels were similar prior to E2/PLAC administration (*z* = 0.17, *p* = .867, *r* = 0.03) and between pre- and post-PLAC drug administration (*z* = −1.46, *p* = .145, *r* = −0.29). Following E2 administration, E2 levels were effectively increased, compared with baseline (*z* = −4.02, *p* < .001, *r* = −0.88) and with PLAC drug administration (*z* = 3.82, *p* < .001, *r* = 0.88). P4 and testosterone levels remained low. For median noncentered hormone levels, see Tables S1 and S2 and Figure S2.

### E2 Increase Is Negatively Associated With Striatum Volume

**Figure 3:**
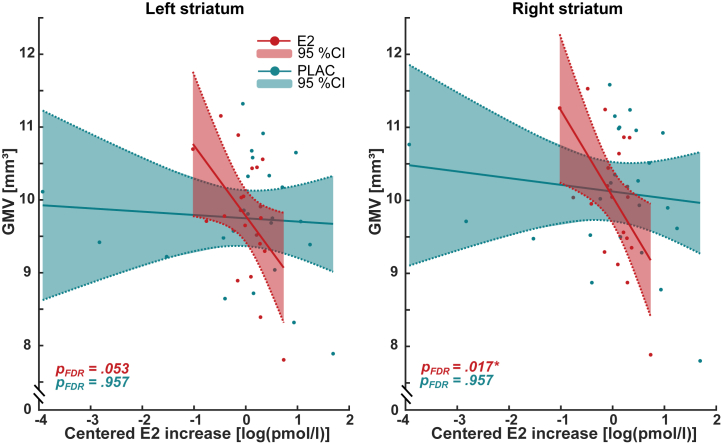
Association between log-centered estradiol (E2) increase (E2Δ) and bilateral striatum gray matter volume (GMV) (mm^3^) during E2 (red) and placebo (PLAC) (blue) administration. The 95% CIs and significance levels are indicated. Note that E2Δ is centered in relation to its mean for each E2 and PLAC condition (0 = mean). ∗*p* < .05. FDR, false discovery rate.

Multiple linear regression analyses revealed that in the E2 drug condition, E2Δ was negatively associated with bilateral striatal GMV (partial *R*^2^_left_ = 0.636, β_left_ = −0.685, *p*_left_ = .013; partial *R*^2^_right_ = 0.694, β_right_ = −0.854, *p*_right_ = .002). However, only the right ventral striatum survived correction for multiple comparisons (*p*_FDR_ = .017). No significant association with striatal GMV was found during the PLAC drug condition (partial *R*^2^_left_ = 0.402, β_left_ = 0.009, *p*_left_ = .908; partial *R*^2^_right_ = 0.403, β_right_ = −0.009, *p*_right_ = .921). The effect of E2Δ on the bilateral striatum was significantly different between the E2 and PLAC conditions (*z*_left_ = −2.676, *p*_left_ = .030; *z*_right_ = −3.374, *p*_right_ = .006), showing a stronger negative association during the E2 drug condition (Figure 3 and Table S4). No associations between E2 or PLAC drug administration were noted in the GMV of the amygdala, hippocampus, or ACC. Statistical parameters are listed in Table S4.

GMV of the amygdala, ACC, hippocampus, and striatum did not differ between drug conditions (*p* > .05) (Table S3).

### Emotion Regulation Trait Affects Striatal GMV Independent of Drug Condition

#### Reappraisal

**Figure 4:**
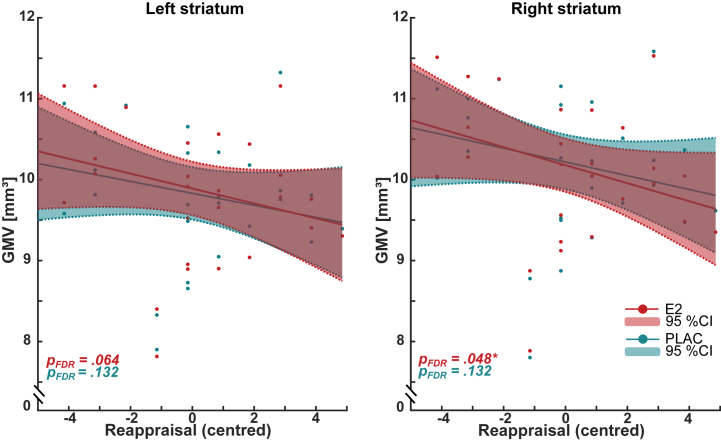
Association between emotion regulation strategy, reappraisal, and bilateral striatum gray matter volume (GMV) (mm^3^) during estradiol (E2) (red) and placebo (PLAC) (blue) administration. The 95% CIs and significance levels are indicated. Note that reappraisal is centered relative to its mean (0 = mean). ∗*p* < .05. FDR, false discovery rate.

Trait reappraisal was negatively associated with GMV of the bilateral striatum in the E2 (partial *R*^2^_left_ = 0.148, β = −0.102, *p*_left_ = .016; partial *R*^2^_right_ = 0.213, β = 0.114, *p*_right_ = .006) and PLAC (partial *R*^2^_left_ = 0.097, β = −0.081, *p*_left_ = .033; partial *R*^2^_right_ = 0.125, β = −0.090, *p*_right_ = .028) drug conditions (Figure 4). Right striatal effects survived correction during the E2 drug condition (*p*_FDR_ = .048). Beta values did not differ significantly between the E2 and PLAC drug conditions (*z*_left_ = −0.390, *p*_left_ = .897; *z*_right_ = −0.454, *p*_right_ = .897), suggesting an equal relationship across conditions (Figure 4 and Table S5). Reappraisal was not significantly associated with GMV of the amygdala, hippocampus, or ACC in either drug condition (see Table S5).

#### Rumination

Trait rumination was not associated with GMV of any ROI during either drug condition (*p* > .05). Parameters are listed in Table S6.

#### Interaction

No significant interactions between E2 and emotion regulation trait appeared (*p* > .05). Complete statistical parameters are listed in Tables S7 and S8.

## Discussion

In the current randomized, placebo-controlled, crossover study, we aimed to identify the rapid effect of E2 on brain plasticity in naturally cycling females. Additionally, we wanted to explore the effect of E2 on brain anatomy in females applying reappraisal and rumination in everyday life. First, we demonstrated that the reliable and rapid increase of E2 levels was negatively associated with right striatal GMV, with the relationship being more pronounced in the E2 drug than in the PLAC drug condition. Second, trait reappraisal use was also negatively associated with right striatal GMV; however, this effect was independent of E2 levels.

### E2 Affects Striatal Volumes

Our data show that the rapid increase in E2, which mimics the preovulatory surge in the middle of the menstrual cycle, impacts brain plasticity particularly in the bilateral striatum: A greater increase in E2 was associated with a greater decrease in GMV. This negative association differed significantly from the PLAC drug condition. Thus, by using a placebo-controlled crossover design during a phase of low P4 and testosterone levels, our findings indicate that elevated E2 levels are associated with decreased GMVs of the striatum. Because endogenous periovulatory surges typically rise quickly over 1 to 2 days (46), the magnitude and timing of the E2 increase in our protocol closely parallel natural patterns, although at somewhat lower levels, supporting the physiological relevance of our manipulation.

Our data align well with findings from the menstrual cycle, where hormone levels naturally fluctuate. The putamen (as part of the dorsal striatum) decreases in size during the preovulatory phase, when E2 levels are high, compared with the luteal phase (10). Similar results were observed by Protopopescu *et al.* (9), who showed decreased right putamen GMV during the late follicular phase (rising E2 levels). This is further supported by studies examining the effects of exogenous sex hormone administration via birth control pills, which typically administer a synthetic estrogen such as ethinyl-E2 (combined with synthetic progestin) for 3 weeks, followed by a 1-week pill-free interval. During this pill-free interval, when hormone levels decline to levels comparable with the early follicular phase (47), caudate GMV (part of the ventral striatum) increased (35). Taken together with the current data, this suggests that the striatum and its subregions are sensitive to fluctuations in both exogenous and endogenous E2 variations.

Animal studies have shown that E2 can rapidly influence the dorsal striatum (48,49) by modulating neuroplastic mechanisms including cellular proliferation (50), dendritic spine density (51), and synaptic plasticity (52). In female Syrian hamsters, E2 administration decreased dendritic spine density and altered cell morphology in the nucleus accumbens (part of the ventral striatum) (53), supporting the idea of localized E2-driven structural remodeling in the striatum.

It is well known that E2 can exert rapid effects via both rapid, nongenomic pathways through membrane-bound G protein–coupled estrogen receptors (within minutes) and genomic pathways, such as transcriptional regulation via nuclear ERs (hours to days) (54). ER subtypes are widely expressed in the brain, including in the striatum (14,15), in place for E2 to locally modulate neural plasticity. Our findings add evidence that such changes can occur rapidly after 24 hours and 12 mg of E2-valerate administration in humans. The volumetric changes may reflect E2-induced alterations in neuronal morphology, dendritic spine density, and shifts in cellular composition (neuron to glia cell ratio) (55, 56, 57). E2 appears to affect different spine types in distinct ways: While spines of pyramidal neurons in the CA1 region of rodent hippocampus increase with E2 administration, certain spine subtypes in the nucleus accumbens were found to be reduced (54). This synaptic pruning may contribute to reduced striatal volumes (55, 56, 57, 58). GMV largely reflects cell bodies (somas) (59). Reductions in striatal volumes have previously been observed with an increase in neuron to glia cell ratio in rats (56). Given that neurons and glia cells differ in soma size (60) and that both neurons and glia cells express ERs (61), such shifts in cellular composition may partially explain the striatal GMV decrease observed in our study.

Despite finding significant effects of E2 on striatum GMV, we did not observe an effect for the ACC, amygdala, or hippocampus. This contrasts somewhat with previous findings showing that ACC volume is negatively correlated with E2 levels during the midluteal menstrual cycle phase (35). Other studies have reported plasticity of the amygdala and hippocampus across different menstrual cycle phases (8, 9, 10,12). This discrepancy is most likely explained by our focus on the effects of rapid E2 increase and, speculatively, a different ratio of ER subtypes. Adult striatal neurons seem to primarily express G protein–coupled ERs (62), whereas cells of the amygdala mainly express nuclear ERs (63). This receptor profile may allow for faster, nongenomic signaling cascades and can account for the fast and immediate effects of E2 that we observed in the striatum, compared with slower, genomic-driven changes. Thus, the striatum seems to have a heightened sensitivity to E2 and rapidly adapts in response to hormonal shifts. Other brain regions may adapt to E2 variations in a slower fashion due to their receptor profile. The striatum is a key subcortical structure involved in stress reactivity, emotion regulation, and reward processing (64, 65, 66). The dorsal striatum, comprising the dorsal caudate and dorsal putamen, receives afferents from the sensorimotor cortex and association cortices (66), supporting cognitive control of emotion and facilitating goal-directed modulation of affective responses (64). The ventral striatum, including the nucleus accumbens and ventral parts of the caudate and putamen, is primarily connected to limbic and orbitofrontal regions (66), enabling evaluation of emotional valence and reward-related processing (64), and is modulated by E2 through enhanced dopaminergic signaling (3). The caudoventral striatum plays a critical role in integrating affective and motivational signals based on its functional and anatomical connections (67). Thus, taken together with our data, this suggests that the striatum is a critical region sensitive to E2 fluctations and that it further drives motoric, sensomotoric, affective, stress, and reward-related behavior.

### Association Between Emotion Regulation and Brain Volume

Our findings indicate that the tendency to use reappraisal or rumination did not impact GMV differently in phases of elevated versus low E2 levels. Although reappraisal was significantly associated with rapidly increasing E2 levels, the effect did not differ significantly between drug conditions. This indicates that there is no reliable evidence for a drug-specific effect of reappraisal, and the observed difference in significance likely reflects variability. No association occurred between GMV and trait use of rumination.

Consistent with our results, Yoon and Jung (30) reported that greater use of reappraisal was related to smaller GMV in the left ventral striatum in a mixed sex sample (not controlling for cycle phase or hormonal contraceptive use). Together these findings support that trait use of reappraisal can contribute to experience-dependent brain plasticity through use of specific cognitive and behavioral patterns (68). More broadly, individual differences in emotion regulation traits have been associated with structural variations in relevant brain areas (34,68).

Taken together with our data, this suggests that affective traits can also shape brain anatomy. In our sample, participants reported more frequent use of reappraisal and less rumination compared with a predominantly female sample reported by Idzadpanah *et al.* (37), potentially explaining the absence of an association between rumination and GMV. Importantly, our sample consisted of healthy young females, with participants excluded in case of moderate to severe depressive symptoms (BDI-II score >20). Prior studies linking rumination to GMV differences often involved clinical populations with higher and more dysfunctional rumination levels (27,69). Thus, the low frequency in rumination in the current sample might have further contributed to the lack of effects for rumination.

Importantly, the impact of trait use of reappraisal on striatal GMV does not seem to be dependent on E2 levels. Thus, E2 levels do not shape striatal volumes differently in individuals using more reappraisal compared with those who apply less reappraisal in everyday life.

### Limitations and Future Directions

By focusing on healthy, naturally cycling females in the early follicular phase of the menstrual cycle, our study was designed to isolate rapid E2-related structural effects while minimizing potential confounding variables (including P4 levels). This targeted approach gives insight into the effects of E2 in healthy females; however, it does not capture the broader hormonal context in which sex hormones may naturally (inter)act (8,26). By applying a within-subject design and controlling for baseline E2 levels (E2Δ), we tried to mitigate some of the naturally occurring hormone fluctuations. Hormonal levels may vary from cycle to cycle depending on the number of antral follicles recruited at the very beginning of the cycle. We also need to consider the individual differences in sensitivity to E2 as metabolic rates may introduce variability in E2 levels across participants. Oral administration of E2-valerate, as administered in the current study, undergoes first-pass metabolism in the gut and liver before it becomes bioavailable (70). Thus, future studies should explore the effect of different routes of E2 administration on GMV. Additionally, future studies should explore the directionality of E2’s effect, as we were not able to directly assess volumetric changes prior to drug administration.

We investigated rapid changes in E2; however, slower and more sustained hormonal changes also occur across the menstrual cycle, during perimenopause, or under hormone therapy. These likely engage distinct neurobiological mechanisms compared with rapid fluctuations (e.g., by gene transcription), which may lead to specific changes in GMV. This warrants a closer investigation of how rapid versus slow E2 dynamics differentially shape brain structure.

### Potential Clinical Implications

The fact that E2 administration can lead to rapid reductions in GMV volume may have implications for mood and mental health. Given that smaller striatal volumes have been linked to better mental health outcomes (69,71), such changes may represent a beneficial neuroplastic response, particularly during hormonally sensitive periods. There is some evidence that E2 treatment reduces perimenopausal depressive symptoms (transdermal) (72), improves mood in females with a history of major depressive disorder (73), and reduces anxiety- and stress-related behaviors in both animal models (74) and naturally cycling females (75). This emphasizes the importance of extending research to diverse female populations, including those diagnosed with affective disorders such as premenstrual dysphoric disorder, where impaired emotion regulation and lower premenstrual E2 levels are associated with greater anxiety and stress (76). Since onset and relapse in females are often related to hormonal fluctuations across the menstrual cycle, pregnancy, the postpartum period, or the transition to menopause (known as perimenopause) (77), understanding individual E2-related brain changes could enable more targeted interventions and turning a window of vulnerability into a window of opportunity. Thus, future research should examine whether females with greater neuroplasticity are also more sensitive to hormonal fluctuations and whether E2-induced striatal volume changes can predict symptom occurrence in those with affective disorders. Such insights could help refine personalized and hormone-informed treatments to improve the quality of life of females across the lifespan.

### Conclusions

Our findings suggest rapid effects of E2 and an association between trait reappraisal use and striatal gray matter volume in healthy, naturally cycling females. Reduced striatal volume was linked to both elevated E2 levels and greater use of reappraisal. This is the first study to assess rapid anatomical changes in the striatum and limbic system resulting from an E2 increase using a within-subject longitudinal design, highlighting fast and efficient neuroplastic adaptations to fluctuating sex hormone levels in healthy females. As structural changes may serve as proxies for functional (78) and behavioral shifts (79), morphological changes of the striatum can subsequently signify meaningful alterations in circuitries associated with emotion regulation and mental health. In this context, E2 may foster structural refinement in key affective circuits and may support mental health across female hormonal transition phases.

## Supplementary Material

Supplemental Methods, Results, Figures S1–S2, Tables S1–S8

Key Resources Table
