# Supplementary material for: Estradiol, Emotion Regulation, and the Limbic System: Effects on Gray Matter Volume
Source: Biol Psychiatry Glob Open Sci. 2026 Feb 17;6(3):100709. doi: 10.1016/j.bpsgos.2026.100709 (PMC13090704; doi:10.1016/j.bpsgos.2026.100709)
Supplement: Supplemental Methods, Results, Figures S1–S2, Tables S1–S8 [file mmc1.pdf]

SUPPLEMENTARY INFORMATION

Oestradiol, Emotion Regulation, and the Limbic System: Effects on Gray Matter Volume

Denninger *et al.*

This document includes supplementary methods, figures S1 & S2, tables S1-S8

Supplementary methods

The regions of interest (ROI) - anterior cingulate cortex (ACC), hippocampus, and striatum – where further dissected into subregions including dorsal, subgenual and pregenual ACC, para- and hippocampus, ventral, and caudoventral striatum (Fig. S1). We then examined the effects of E2 increase (E2Δ) and trait emotion regulation strategies on the GMV of these subregions. Due to the exploratory nature of these analyses, no correction for multiple comparisons was applied.

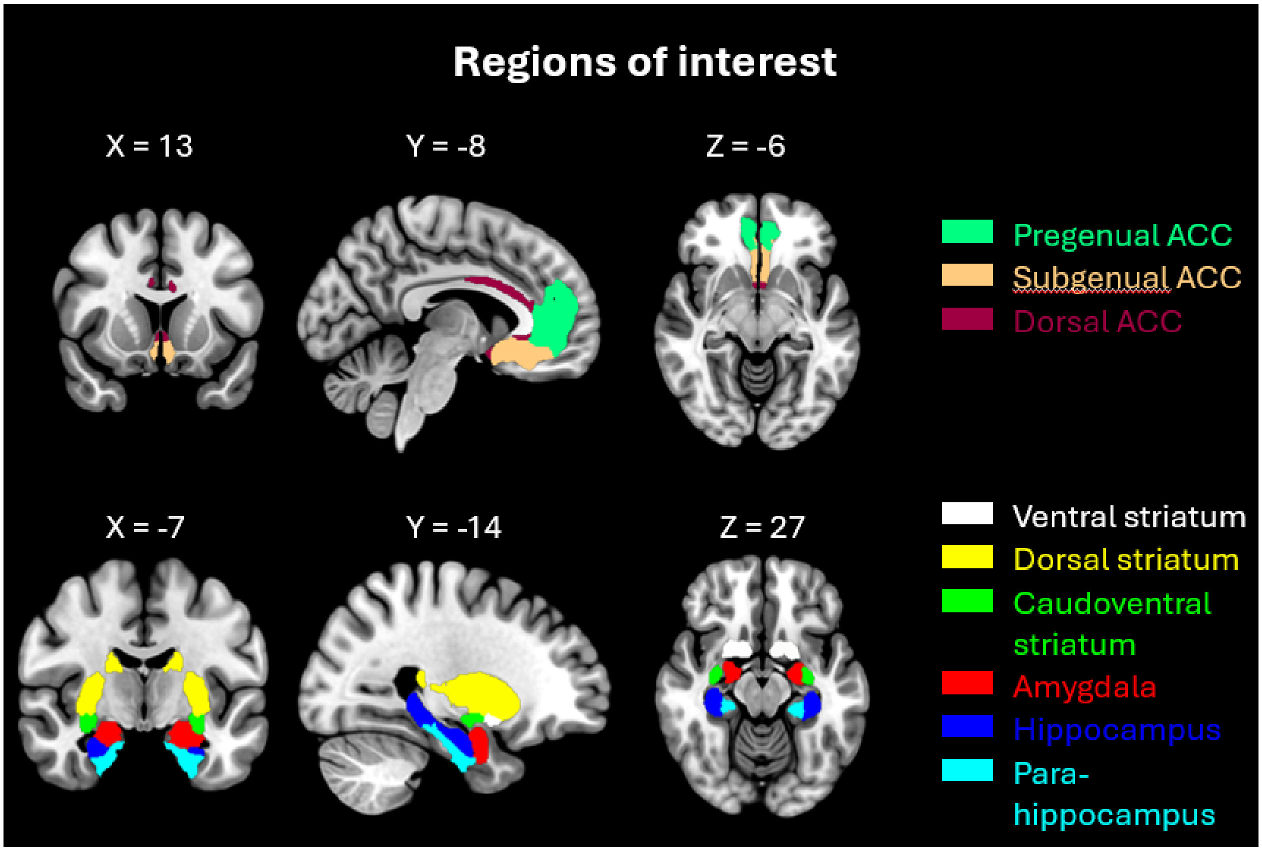

Fig. S1 | Extended region of interest (ROI) in MNI-space.

Supplementary results

E2 increase: Similar to the whole striatum, exploratory analysis of striatal subregions showed that E2 increase (E2Δ) was negatively associated with right ventral (partial-R^2_{right}=.558, β_{right}=-.110, p_{right}=.007), right caudoventral (partial-R^2_{right}=.431, β_{right}=-.044, p_{right}=.012) and bilateral dorsal striatum (partial-R^2_{left}=.630, β_{left}=-.584, p_{left}=.019; partial-R^2_{right}=.696, β_{right}=-.073, p_{right}=.003) (Tab.S4). Slopes differed between drug conditions in the right ventral (p = .016) and caudoventral (p = .001) as well as bilateral dorsal striatum (p_{left} = .012, p_{right} = .002).

Reappraisal: Reappraisal was negatively associated with right ventral (E2: partial-R^2_{right}=.133, β_{right}=-.012, p_{right}=.025; PLAC: partial-R^2_{right}=.159, β_{right}=-.014, p_{right}=.038) and bilateral caudoventral (E2: partial-R^2_{left}=.154, β_{left}=-.005, p_{left}=.012; partial-R^2_{right}=.279, β_{right}=-.006, p_{right}=.003; PLAC: partial-R^2_{left}=.149, β_{left}=-.004, p_{left}=.047) and dorsal (E2: partial-R^2_{left}=.163, β_{left}=-.092, p_{left}=.014; partial-R^2_{right}=.206, β_{right}=-.085, p_{right}=.016; PLAC: partial-R^2_{left}=.094, β_{left}=-.07, p_{left}=.036; partial-R^2_{right}=.112, β_{right}=-.071, p_{right}=.040) striatum GMV. However, slopes did not differ between the E2 and PLAC drug conditions (Tab.S5).

Rumination: Left para-hippocampus was positively associated with trait use of rumination during the E2 drug condition (partial-R^2_{right}=.198, β_{right}=-.022, p_{right}=.038). Slopes did not differ between E2 and PLAC drug conditions (Tab S6).

Supplementary figures & tables

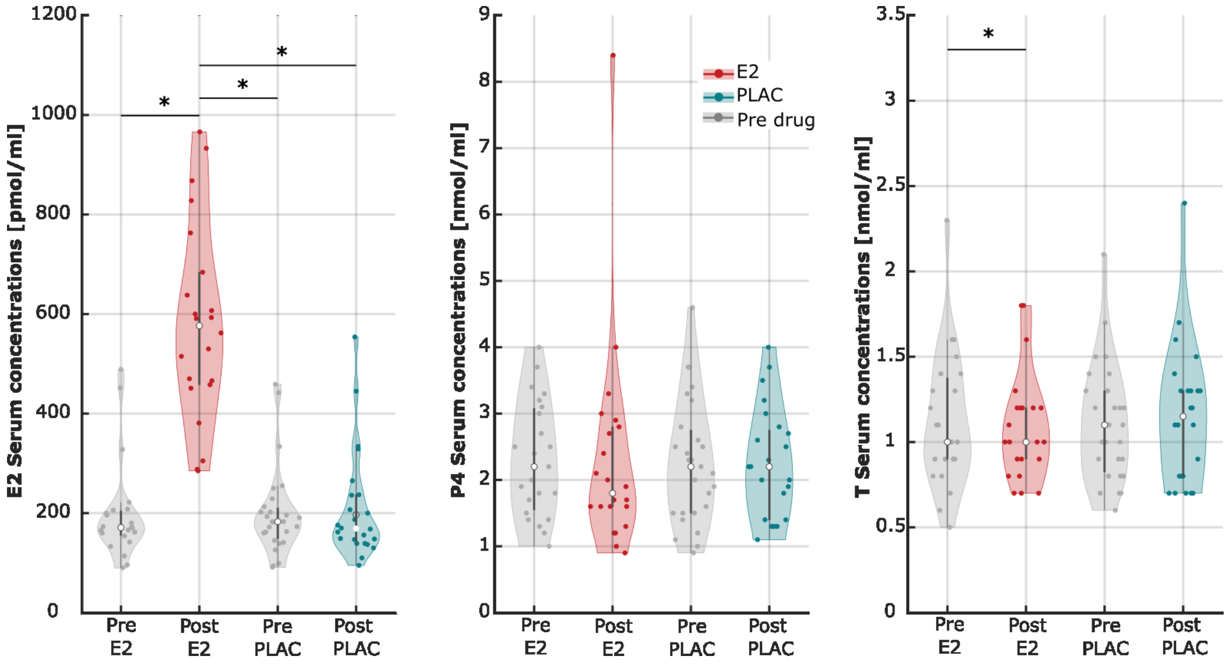

Fig. S2 | Serum hormone concentrations of E2 [pmol/l], progesterone (P4) [nmol/l], testosterone (T) [nmol/l] pre (grey) and post E2 (red) and post PLAC (blue) administration. Significance is indicated with an asterisk.

Tab. S1 | Median serum hormone concentration of E2 [pmol/l], P4 [nmol/l], T [nmol/l] pre and post drug administration (E2/PLAC) and significance levels of E2, P4 and T concentrations comparing drug conditions (Z-statistics, effect size). Significant differences are labelled with an asterisk.

	**PRE E2**<br>Median *(IQR)*<br>p *(Z,r)*	**POST E2**<br>Median *(IQR)*<br>p *(Z,r)*	**PRE PLAC**<br>Median *(IQR)*<br>p *(Z,r)*	**POST PLAC**<br>Median *(IQR)*<br>p *(Z,r)*
**E2 [pmol/l]**	**171.0 *(155.5, 204.5)***	**576.5 *(458.0, 684.0)***	**183.0 *(148.75, 210.25)***	**169.0 *(143.9, 236.5)***
PRE E2:		< .001* *(-4.02, -.88)*	.867 *(.17, .03)*	.689 *(-.40, -.09)*
POST E2:			< .001* *(4.12, .84)*	< .001* *(3.82, .88)*
PRE PLAC:				.145 *(1.46, .30)*
**P4 [nmol/l]**	**2.2 *(1.55, 3.075)***	**1.8 *(1.6, 2.8)***	**2.2 *(1.5, 2.75)***	**2.2 *(1.4, 2.75)***
PRE E2:		.199 *(1.28, .28)*	.961 *(-.05, -.01)*	.903 *(-.12, -.03)*
POST E2:			.197 *(-1.29, -.27)*	.409 *(-.83, -.19)*
PRE PLAC:				.379 (.88, .18)
**T [nmol/l]**	**1.0 *(0.9, 1.375)***	**1.0 *(0.9, 1.2)***	**1.1 *(0.825, 1.3)***	**1.15 *(0.8, 1.3)***
PRE E2:		.038* *(2.07, .45)*	.734 *(-.34, -07)*	.536 *(-.62, -.14)*
POST E2:			.134 *(-1.50, -.32)*	.134 *(-1.50, -.34)*
PRE PLAC:				.238 *(-1.18, -.24)*

Tab. S2 | Median E2 increase in [pmol/l] and centred log transformed values for E2 and PLAC drug condition. Inter quartile range is indicated.

E2 adminsitration		PLAC administration	
ΔE2 [pmol/l]	Centred & log ΔE2 [log(pmol/l)]	ΔE2 [pmol/l]	Centred & log ΔE2 [log(pmol/l)]
576.5 [458, 684]	.050 *[-.180, .221]*	1.69 *[143, 236.5]*	-.115 *[-.282, .222]*

Tab. S3 | Comparison of grey matter volume in mm³ between E2 and PLAC drug condition. Median volumes and inter quartile range, as well as statistical parameters of a sign rank test are indicated.

	**E2 administration** Median volume [mm³]	**PLAC administration** Median volume [mm³]	p-value	Z-value
**Amygdala (L)**	2.398 [.205]	2.352 [.214]	.203	1.273
**Amygdala (R)**	2.386 [.251]	2.355 [.276]	.280	1.081
**ACC (L)**	7.289 [1.037]	7.289 [1.197]	.361	.913
**ACC (R)**	7.137 [.899]	7.125 [1.003]	.701	.384
**Hippocampus (L)**	5.754 [.541]	5.774 [.512]	.923	.096
**Hippocampus (R)**	5.569 [.394]	5.527 [.290]	.943	.072
**Striatum (L)**	9.863 [1.119]	9.792 [1.197]	.164	1.393
**Striatum (R)**	10.128 [1.305]	10.239 [1.403]	1	0

R = right, L = left

Effects of E2 increase on limbic GMV

Tab. S4 | Statistical parameters from robust mixed linear regression analysing the relationship between oestradiol (E2) increase and regional gray matter volume (GMV) under E2 and placebo (PLAC) conditions. Slope coefficients (β), standard errors (SE), t-statistics, and uncorrected as well as false discovery rate (FDR)-corrected p-values for the association between E2 increase and GMV across both drug conditions are reported. Additionally, p-values from Z-transformed slope comparisons (E2 vs. PLAC) are included to assess condition-specific differences in association strength. Significant associations ($p < .05$) are marked with an asterisk (*). Under the E2 condition, E2 increase was significantly negatively associated with GMV in the right ventral and caudoventral striatum, as well as bilaterally in the dorsal striatum, as well as overall striatum. These associations were not observed during the PLAC condition, and slope comparisons between conditions indicated significant differences in these regions.

**Left amygdala**													
	**E2 condition**						**PLAC condition**						**Z test**
	β	SE	t-stat	p_{uncor}	p_{FDR}	partial R^2	β	SE	t-stat	p_{uncor}	p_{FDR}	partial R^2	p_{FDR}
**ΔE2**	-.028	.067	-.423	.678	.880	.304	.017	.024	.719	.480	.932	.142	.892
**TIV**	.001	<.001	5.172	<.001*	<.001*	.648	.001	< .001	4.917	<.001*	<.001	.586	.961
**Age**	-.003	.009	-.339	.739	.884	.008	-.003	.011	-.256	.801	.801	.002	.981
**Model**	N = 21, R^2 = .119, F = 10.5, p < .001*						N = 24, R^2 = .562, F= 8.6, p < .001*						

**Right amygdala**													
	**E2 condition**						**PLAC condition**						**Z test**
	β	SE	t-stat	p_{uncor}	p_{FDR}	partial R^2	β	SE	t-stat	p_{uncor}	p_{FDR}	partial R^2	p_{FDR}
**ΔE2**	-.081	.079	-1.019	.322	.880	.336	.020	.025	.0804	.431	.932	.198	.602
**TIV**	.002	<.001	5.165	<.001*	<.001*	.627	.002	<.001	5.662	<.001*	<.001	.650	.961
**Age**	-.001	.011	-.114	.911	.911	.006	-.006	.012	-.511	.615	.801	.009	.981
**Model**	N = 21, R^2 = .658, F = 10.9, p < .001*						N = 24, R^2 = .634, F = 11.5, p < .001*						

**Left ACC**													
	**E2 condition**						**PLAC condition**						**Z test**
	β	SE	t-stat	p_{uncor}	p_{FDR}	partial R^2	β	SE	t-stat	p_{uncor}	p_{FDR}	partial R^2	p_{FDR}

**ΔE2**	-.130	.266	-.487	.632	.880	.394	.005	.083	.054	.957	.957	.302	.892
**TIV**	.007	.001	6.206	<.001*	<.001*	.714	.006	.001	6.681	<.001*	<.001*	.719	.961
**Age**	-.077	-.039	-2.011	.060	.484	.196	-.073	.039	-1.859	.078	.622	.168	.981
**Model**	N = 21, R^2 = .763, F = 18.3, p < .001*						N = 24, R^2 = .733, F = 18.3, p < .001*						

**Right ACC**													
	**E2 condition**						**PLAC condition**						**Z test**
	β	SE	t-stat	p_{uncor}	p_{FDR}	partial R^2	β	SE	t-stat	p_{uncor}	p_{FDR}	partial R^2	p_{FDR}
**ΔE2**	-.156	.268	-.058	.569	.880	.291	-.038	.070	-.558	.583	.932	.473	.892
**TIV**	.006	.001	5.930	<.001*	<.001*	.680	.006	.001	7.836	<.001*	<.001	.782	.961
**Age**	-.035	.039	-.906	.378	.884	.036	-.028	.033	-.839	.411	.801	.040	.981
**Model**	N = 21, R^2 = .721, F = 14.7, p < .001*						N = 24, R^2 = .772, F = 22.6, p < .001*						

**Left perigenual ACC**													
	**E2 condition**						**PLAC condition**						**Z test**
	β	SE	t-stat	p_{uncor}	p_{FDR}	partial R^2	β	SE	t-stat	p_{uncor}	p_{FDR}	partial R^2	p
**ΔE2**	.015	.190	.078	.987		.371	-.001	.063	-.017	.987		.193	.937
**TIV**	.005	< .001	6.041	<.001*		.720	.004	< .001	5.972	<.001*		.678	.777
**Age**	-.059	.027	-2.132	.071		.233	-.057	.030	-1.904	.071		.172	.962
**Model**	N = 21, R^2 = .752, F = 17.2, p < .001*						N = 24, R^2 = .694, F = 15.1, p < .001*						

**Right perigenual ACC**													
	**E2 condition**						**PLAC condition**						**Z test**
	β	SE	t-stat	p_{uncor}	p_{FDR}	partial R^2	β	SE	t-stat	p_{uncor}	p_{FDR}	partial R^2	p
**ΔE2**	-.045	.194	-.235	.817		.238	-.033	.0517	-.642	.528		.459	.208
**TIV**	.004	.001	5.450	<.001*		.635	0.004	0.001	7.1257	<.001*		.757	.709

**Age**	-.028	.028	-.996	.333		.038	-.023	.024	-.932	.362		.044	1
**Model**	N = 21, R^2 = .686, F = 12.4, p < .001*						N = 24, R^2 = .741, F = 19.1, p < .001*						

**Left subgenual ACC**													
	**E2 condition**						**PLAC condition**						**Z test**
	β	SE	t-stat	p_{uncor}	p_{FDR}	partial R^2	β	SE	t-stat	p_{uncor}	p_{FDR}	partial R^2	p
**ΔE2**	-.111	.087	-1.27	.220		.329	.006	.025	.246	.808		.374	.196
**TIV**	.002	< .001	4.686	<.001*		.534	.002	<.001	5.305	<.001*		.625	.823
**Age**	-.013	.013	-1.025	.320		.040	-.012	.012	-1.018	.321		.049	.966
**Model**	N = 21, R^2 = .623, F = 10.6, p < .001*						N = 24, R^2 = .617, F = 10.7, p < .001*						

**Right subgenual ACC**													
	**E2 condition**						**PLAC condition**						**Z test**
	β	SE	t-stat	p_{uncor}	p_{FDR}	partial R^2	β	SE	t-stat	p_{uncor}	p_{FDR}	partial R^2	p
**ΔE2**	-.087	.065	-1.336	.200		.397	-.002	.019	-.081	.936		.370	.623
**TIV**	.002	<.001	6.123	<.001*		.704	.002	<.001	6.792	<.001*		.731	.743
**Age**	-.008	1	-.904	.379		.031	-.009	.009	-.951	.353		.044	.674
**Model**	N = 21, R^2 = .747, F = 16.8, p < .001*						N = 24, R^2 = .720, F = 17.1, p < .001*						

**Left dorsal ACC**													
	**E2 condition**						**PLAC condition**						**Z test**
	β	SE	t-stat	p_{uncor}	p_{FDR}	partial R^2	β	SE	t-stat	p_{uncor}	p_{FDR}	partial R^2	p
**ΔE2**	-.028	.027	-1.040	.313		.389	.002	.008	.296	.771		.318	.280
**TIV**	< 001	<.001	4.119	.001*		.508	<.001	<.001	5.241	<.001*		.601	.845
**Age**	-.007	.004	-1.74	.099		.146	-.006	.004	-1.533	.141		.130	.839
**Model**	N = 21, R^2 = .628, F = 9.6, p < .001*						N = 24, R^2 = .628, F = 11.3, p < .001*						

**Right dorsal ACC**													
	**E2 condition**						**PLAC condition**						**Z test**
	β	SE	t-stat	p_{uncor}	p_{FDR}	partial R^2	β	SE	t-stat	p_{uncor}	p_{FDR}	partial R^2	p
**ΔE2**	.015	.037	.393	.699		.384	-.005	.012	-.382	.706		.165	.984
**TIV**	<.001	<.001	4.093	.001*		.543	<.001	<.001	3.947	.001*		.463	.595
**Age**	-.001	.005	-.267	.792		.003	.002	.006	.326	.748		.003	.948
**Model**	N = 21, R^2 = .522, F = 6.2, p = .005*						N = 24, R^2 =.445, F = 5.3, p =.007*						

**Left total hippocampus**													
	**E2 condition**						**PLAC condition**						**Z test**
	β	SE	t-stat	p_{uncor}	p_{FDR}	partial R^2	β	SE	t-stat	p_{uncor}	p_{FDR}	partial R^2	p_{FDR}
**ΔE2**	.029	.191	.153	.880	.880	.254	-.084	.051	1.666	.112	.890	.195	.892
**TIV**	.003	.001	4.011	.001*	.001*	.530	.003	.001	4.946	<.001*	<.001*	.578	.961
**Age**	-.008	.028	-.293	.773	.884	.004	-.013	.024	-.533	.600	.801	.009	.981
**Model**	N = 21, R^2 = .516, F = 6.0, p = .005*						N = 24, R^2 = .587, F= 9.5, p < .001*						

**Right total hippocampus**													
	**E2 condition**						**PLAC condition**						**Z test**
	β	SE	t-stat	p_{uncor}	p_{FDR}	partial R^2	β	SE	t-stat	p_{uncor}	p_{FDR}	partial R^2	p_{FDR}
**ΔE2**	.044	.197	.223	.826	.880	.153	.040	.054	.731	.473	.932	.150	.892
**TIV**	.003	.001	3.958	.001*	.001*	.538	.003	.001	4.569	<.001*	<.001*	.563	.961
**Age**	-.015	.029	-.528	.604	.884	.012	-.014	.026	-.553	.586	.801	.013	.981
**Model**	N = 21, R^2 = .518, F = 6.1, p = .005*						N = 24, R^2 = .535, F= 7.7, p = .001*						

Left hippocampus

	E2 condition						PLAC condition						Z test
	β	SE	t-stat	p_{uncor}	p_{FDR}	partial R^2	β	SE	t-stat	p_{uncor}	p_{FDR}	partial R^2	p
**ΔE2**	.014	.095	.148	.884		.314	.037	.027	1.385	.181		.201	.813
**TIV**	.002	<.001	4.963	<.001*		.611	.002	<.001	5.076	<.001*		.550	.511
**Age**	-.004	.014	-.317	.755		.003	-.012	.013	-1.041	.310		.032	.634
**Model**	N = 21, R^2 = .620, F = 9.2, p < .001*						N = 24, R^2 = .604, F = 10.2, p < .001*						

Right hippocampus

	E2 condition						PLAC condition						Z test
	β	SE	t-stat	p_{uncor}	p_{FDR}	partial R^2	β	SE	t-stat	p_{uncor}	p_{FDR}	partial R^2	p
**ΔE2**	.016	.119	.134	.895		.118	.013	.035	.379	.709		.028	.921
**TIV**	.002	<.001	3.942	.001*		.538	.002	<.001	3.829	.001*		.484	.942
**Age**	<.001	.0172	.035	.972		<.001	<.001	.017	-.058	.954		<.001	.883
**Model**	N = 21, R^2 = .497, F = 5.6, p = .007*						N = 24, R^2 = .433, F = 5.1, p = .009*						

Left para-hippocampus

	E2 condition						PLAC condition						Z test
	β	SE	t-stat	p_{uncor}	p_{FDR}	partial R^2	β	SE	t-stat	p_{uncor}	p_{FDR}	partial R^2	p
**ΔE2**	.025	.112	.224	.826		.186	.042	.029	1.443	.165		.203	.884
**TIV**	.001	<.001	2.693	.015*		.341	.001	<.001	4.049	.001*		.485	.809
**Age**	-.003	.016	-.198	.845		.004	.004	.014	.295	.771		<.001	.733
**Model**	N = 21, R^2 = .323, F = 2.7, p = .078						N = 24, R^2 = .482, F = 6.2, p = .004*						

Right para-hippocampus

	E2 condition						PLAC condition						Z test
	β	SE	t-stat	p_{uncor}	p_{FDR}	partial R^2	β	SE	t-stat	p_{uncor}	p_{FDR}	partial R^2	p

**ΔE2**	.036	.094	.384	.706		.221	.026	.027	.98	.335		.318	.387
**TIV**	.001	<.001	3.391	.004*		.447	.001	<.001	4.307	<.001*		.535	.675
**Age**	-.014	.014	-1.027	.319		.056	-.011	.013	-.893	.383		.051	.968
**Model**	N = 21, R^2 = .472, F = 5.1, p = .011*						N = 24, R^2 = .521, F = 7.2, p = .002*						

Left striatum

	**E2 condition**						**PLAC condition**						**Z test**
	β	SE	t-stat	p_{uncor}	p_{FDR}	partial R^2	β	SE	t-stat	p_{uncor}	p_{FDR}	partial R^2	p_{FDR}
**ΔE2**	-.685	.247	-2.769	.013*	.053	.636	.009	.078	.117	.908	.957	.402	.030*
**TIV**	.005	.001	5.410	<.001*	<.001*	.575	.007	.001	7.413	<.001*	<.001	.629	.961
**Age**	-.027	.036	.749	.464	.884	.103	-.011	.037	-.301	.767	.801	.136	.981
**Model**	N = 21, R^2 = .742, F = 16.3, p < .001*						N = 24, R^2 = .745, F= 19.5, p < .001*						

Right striatum

	**E2 condition**						**PLAC condition**						**Z test**
	β	SE	t-stat	p_{uncor}	p_{FDR}	partial R^2	β	SE	t-stat	p_{uncor}	p_{FDR}	partial R^2	p_{FDR}
**ΔE2**	-.854	.236	-3.625	.002*	.017*	.694	-.009	.085	-.100	.921	.957	.403	.006*
**TIV**	.006	.001	6.334	<.001*	<.001*	.671	.007	.001	6.857	<.001*	<.001	.668	.961
**Age**	-.021	.034	-6.09	.551	.884	.086	-.043	.040	-1.079	.294	.801	.199	.981
**Model**	N = 21, R^2 = .803, F = 23.1, p < .001*						N = 24, R^2 = .729, F= 17.9, p < .001*						

Left ventral striatum

	**E2 condition**						**PLAC condition**						**Z test**
	β	SE	t-stat	p_{uncor}	p_{FDR}	partial R^2	β	SE	t-stat	p_{uncor}	p_{FDR}	partial R^2	p
**ΔE2**	-.100	.045	-2.228	.040*		.459	-.009	.015	-.584	.566		.263	.052
**TIV**	<.001	<.001	4.103	.001*		.520	<.001	<.001	4.896	<.001*		.614	.766

**Age**	-.004	.007	-.632	.536		.020	-.004	.007	-.569	.576		.031	.982
**Model**	N = 21, R^2 = .631, F = 9.7, p < .001*						N = 24, R^2 = .575, F = 9.0, p< .001*						

**Right ventral striatum**													
	**E2 condition**						**PLAC condition**						**Z test**
	β	SE	t-stat	p_{uncor}	p_{FDR}	partial R^2	β	SE	t-stat	p_{uncor}	p_{FDR}	partial R^2	p
**ΔE2**	-.110	.036	-3.099	.007*		.558	-.016	.016	-1.044	.309		.268	.016*
**TIV**	<.001	<.001	5.721	<.001*		.668	<.001	<.001	4.423	<.001*		.535	.897
**Age**	-.002	.005	-.358	.724		.020	-0.009	0.007	-1.218	.238		.087	.429
**Model**	N = 21, R^2 = .760, F = 18.0, p < .001*						N = 24, R^2 = .569, F = 8.8, p < .001*						

**Left dorsal striatum**													
	**E2 condition**						**PLAC condition**						**Z test**
	β	SE	t-stat	p_{uncor}	p_{FDR}	partial R^2	β	SE	t-stat	p_{uncor}	p_{FDR}	partial R^2	p
**ΔE2**	-.584	.226	-2.592	.019*		.630	.009	.067	.138	.892		.414	.012*
**TIV**	.004	<.001	4.669	<.001*		.527	.005	<.001	7.082	<.001*		.598	.300
**Age**	-.033	.033	-.995	.335		.108	-.011	.032	-.359	.724		.144	.644
**Model**	N = 21, R^2 = .699, F = 13.1, p < .001*						N = 24, R^2 = .729, F = 18.0, p < .001*						

**Right dorsal striatum**													
	**E2 condition**						**PLAC condition**						**Z test**
	β	SE	t-stat	p_{uncor}	p_{FDR}	partial R^2	β	SE	t-stat	p_{uncor}	p_{FDR}	partial R^2	p
**ΔE2**	-.073	.211	-3.465	.003*		.696	-.029	.075	-.389	.701		.424	.002*
**TIV**	.005	<.001	5.696	<.001*		.648	.005	<.001	6.312	<.001*		.664	.615
**Age**	-.027	.031	-.877	.393		.095	-.070	0.036	-1.965	.064		.208	.359
**Model**	N = 21, R^2 = .778, F = 19.8, p < .001*						N = 24, R^2 = .430, F = 17.1, p < .001*						

Left caudoventral striatum													
	**E2 condition**						**PLAC condition**						**Z test**
	β	SE	t-stat	p_{uncor}	p_{FDR}	partial R²	β	SE	t-stat	p_{uncor}	p_{FDR}	partial R²	p
**ΔE2**	-.022	.016	-1.338	.198		.347	.006	.004	1.208	.174		.268	.097
**TIV**	<.001	y.001	3.996	.001*		.459	<.001	<.001	5.209	<.001*		.583	.995
**Age**	<.001	.002	-.068	.947		.018	-.002	.002	-1.009	.325		.083	.539
**Model**	N = 21, R² = .560, F = 7.2, p < .001*						N = 24, R² = .615, F = 10.6, p < .001*						

Right caudoventral striatum													
	**E2 condition**						**PLAC condition**						**Z test**
	β	SE	t-stat	p_{uncor}	p_{FDR}	partial R²	β	SE	t-stat	p_{uncor}	p_{FDR}	partial R²	p
**ΔE2**	-.044	0.015	-2.831	.012*		.431	.009	.005	1.701	.104		.192	.001*
**TIV**	<.001	<.001	2.972	.009*		.376	<.001	<.011	5.059	<.001*		.583	.199
**Age**	<.001	.002	-.429	.673		.007	-.003	.002	-1.320	.202		.103	.502
**Model**	N = 21, R² = .567, F = 7.4, p =.002*						N = 24, R² = .616, F = 10.7, p < .001*						

Effects of trait emotion regulation on limbic GMV

Reappraisal

Tab. S5 | Statistical parameters from robust mixed linear regression analysing the relationship between emotion regulation strategy (trait reappraisal and rumination) and regional gray matter volume (GMV) under E2 and placebo (PLAC) conditions. Slope coefficients (β), standard errors (SE), t-statistics, and uncorrected as well as false discovery rate (FDR)-corrected p-values for the association between E2 increase and GMV across both drug conditions are reported. Additionally, p-values from Z-transformed slope comparisons (E2 vs. PLAC) are included to assess condition-specific differences in association strength. Significant associations (p < .05) are marked with an asterisk (*). Trait reappraisal was negatively associated with right ventral and bilateral caudoventral and dorsal striatum GMV. However, slopes did not differ between E2 and PLAC drug condition.

**Left amygdala**													
	**E2 condition**						**PLAC condition**						**Z test**
	β	SE	t-stat	p_{uncor}	p_{FDR}	partial R^2	β	SE	t-stat	p_{uncor}	p_{FDR}	partial R^2	p_{FDR}
**Reappraisal**	-.003	.009	-.370	.715	.944	.006	-.014	.010	-1.422	.169	.450	.089	.897
**TIV**	.001	<.001	5.386	<.001*	.001*	.602	.001	<.001	5.086	<.001*	.050*	.570	.999
**Age**	.005	.008	.655	.519	.692	.016	.006	.009	.751	.460	.712	.025	.949
**Model**	N = 27, R^2 = .561, F = 9.8, p < .001*						N = 27, R^2 = .542, F= 9.1, p < .001*						

**Right amygdala**													
	**E2 condition**						**PLAC condition**						**Z test**
	β	SE	t-stat	p_{uncor}	p_{FDR}	partial R^2	β	SE	t-stat	p_{uncor}	p_{FDR}	partial R^2	p_{FDR}
**Reappraisal**	-.004	.011	-.397	.695	.944	<.001	-.006	.011	-.593	.559	.745	.014	.897
**TIV**	.002	<.001	5.555	<.001*	<.001*	.561	.001	<.001	5.138	<.001*	.050*	.588	.999
**Age**	.017	.010	1.199	.243	.692	.022	.007	.010	.667	.511	.712	.015	.949
**Model**	N = 27, R^2 = .574, F = 10.3, p < .001*						N = 27, R^2 = .538, F = 8.9, p < .001*						

**Left ACC**													
	**E2 condition**						**PLAC condition**						**Z test**
	β	SE	t-stat	p_{uncor}	p_{FDR}	partial R^2	β	SE	t-stat	p_{uncor}	p_{FDR}	partial R^2	p_{FDR}

**Reappraisal**	-.005	.039	-.140	.890	.944	.001	-.033	.039	-.860	.399	.638	.042	.897
**TIV**	.006	.001	5.545	<.001*	.020*	.607	.006	.001	5.809	<.001*	.013*	.631	.999
**Age**	-.051	.035	-1.449	.161	.692	.091	-.057	.035	-1.625	.118	.712	.117	.949
**Model**	N = 27, R^2 = .626, F = 12.9, p < .001*						N = 27, R^2 = .653, F= 14.5, p < .001*						

**Right ACC**													
	**E2 condition**						**PLAC condition**						**Z test**
	β	SE	t-stat	p_{uncor}	p_{FDR}	partial R^2	β	SE	t-stat	p_{uncor}	p_{FDR}	partial R^2	p_{FDR}
**Reappraisal**	-.023	.037	-.625	.538	.944	.023	-.036	.035	-1.045	.307	.614	.067	.897
**TIV**	.006	.001	5.873	<.001*	.015*	.626	.006	.001	6.370	<.001*	.005*	.643	.999
**Age**	-.016	.033	-.487	.631	.721	.009	-.020	.031	-.632	.534	.903	.013	.949
**Model**	N = 27, R^2 = .624, F = 12.7, p < .001*						N = 27, R^2 = .665, F = 15.2, p < .001*						

**Left perigenual ACC**													
	**E2 condition**						**PLAC condition**						**Z test**
	β	SE	t-stat	p_{uncor}	p_{FDR}	partial R^2	β	SE	t-stat	p_{uncor}	p_{FDR}	partial R^2	p
**Reappraisal**	-.001	.037	-.026	.979		<.001	-.019	.027	-.698	.492		.028	.635
**TIV**	.004	.001	5.443	<.001*		.612	.004	.001	5.40	<.001*		.623	.893
**Age**	-.043	.025	-1.729	.097		.135	-.045	.025	-1.822	.082		.145	.949
**Model**	N = 27, R^2 = .629, F = 13.0, p < .001*						N = 27, R^2 = .648, F = 14.1, p < .001*						

**Right perigenual ACC**													
	**E2 condition**						**PLAC condition**						**Z test**
	β	SE	t-stat	p_{uncor}	p_{FDR}	partial R^2	β	SE	t-stat	p_{uncor}	p_{FDR}	partial R^2	p
**Reappraisal**	-.017	.026	-.644	.526		.031	-.025	.025	-1.012	.322		.077	.813
**TIV**	.004	.001	5.532	<.001*		.608	.004	.001	5.958	<.001*		.618	.888

**Age**	-.015	.024	-.636	.531		.015	-.016	.023	-.718	.480		.014	.969
**Model**	N = 27, R^2 = .601, F = 11.5, p < .001*						N = 27, R^2 = .638, F = 13.5, p < .001*						
**Left subgenual ACC**													
	**E2 condition**						**PLAC condition**						**Z test**
	β	SE	t-stat	p_{uncor}	p_{FDR}	partial R^2	β	SE	t-stat	p_{uncor}	p_{FDR}	partial R^2	p
**Reappraisal**	.002	.013	.168	.868		<.001	-.006	.012	-.466	.646		.016	.658
**TIV**	.001	<.001	4.203	<.001*		.455	.001	< .001	4.474	<.001*		.470	.987
**Age**	-.005	.011	-.407	.688		.005	-.009	.011	-.809	.427		.022	.796
**Model**	N = 27, R^2 = .462, F = 6.6, p = .002*						N = 27, R^2 = .507, F = 7.9, p < .001*						
**Right subgenual ACC**													
	**E2 condition**						**PLAC condition**						**Z test**
	β	SE	t-stat	p_{uncor}	p_{FDR}	partial R^2	β	SE	t-stat	p_{uncor}	p_{FDR}	partial R^2	p
**Reappraisal**	-.003	.009	-.363	.720		.003	-.005	.009	-.570	.574		.025	.892
**TIV**	.001	<.001	5.516	<.001*		.602	.001	<.001	5.764	<.001*		.607	.960
**Age**	-.004	.008	-.507	.617		.011	-.008	.008	-.992	.332		.033	.744
**Model**	N = 27, R^2 = .595, F = 11.3, p < .001*						N = 27, R^2 = .629, F = 13, p < .001*						
**Left dorsal ACC**													
	**E2 condition**						**PLAC condition**						**Z test**
	β	SE	t-stat	p_{uncor}	p_{FDR}	partial R^2	β	SE	t-stat	p_{uncor}	p_{FDR}	partial R^2	p
**Reappraisal**	-.006	.004	-1.630	.117		.127	-.008	.003	-2.256	.034*		.228	.785
**TIV**	<.001	<.001	4.209	<.001*		.475	< .001	<.001	4.946	<.001*		.545	.902
**Age**	-.004	.004	-1.133	.269		.064	-.004	.003	-1.247	.225		.078	.981
**Model**	N = 27, R^2 = .516, F = 8.2, p < .001*						N = 27, R^2 = .601, F = 11.5, p < .001*						

**Right dorsal ACC**													
	**E2 condition**						**PLAC condition**						**Z test**
	β	SE	t-stat	p_{uncor}	p_{FDR}	partial R^2	β	SE	t-stat	p_{uncor}	p_{FDR}	partial R^2	p
**Reappraisal**	-.002	.005	-.360	.722		.009	-.003	.005	-.602	.554		.023	.864
**TIV**	<.001	<.001	3.610	.002*		.394	.001	<.001	3.645	.001*		.403	.977
**Age**	.002	.005	.502	.621		.011	.003	.005	.607	.550		.016	.940
**Model**	N = 27, R^2 = .365, F = 4.4, p = .014*						N = 27, R^2 =.370, F = 4.5, p =.013*						

**Left total hippocampus**													
	**E2 condition**						**PLAC condition**						**Z test**
	β	SE	t-stat	p_{uncor}	p_{FDR}	partial R^2	β	SE	t-stat	p_{uncor}	p_{FDR}	partial R^2	p_{FDR}
**Reappraisal**	.002	.026	.071	.944	.944	<.001	-.002	.023	-.115	.910	.952	<.001	.897
**TIV**	.003	.001	3.902	.001*	.717	.479	.003	.001	4.427	<.001*	.222	.517	.999
**Age**	.018	.023	.762	.454	.692	.032	.014	.021	.670	.510	.712	.022	.949
**Model**	N = 27, R^2 = .400, F = 5.1, p = .007*						N = 27, R^2 = .462, F= 6.6, p = .002*						

**Right total hippocampus**													
	**E2 condition**						**PLAC condition**						**Z test**
	β	SE	t-stat	p_{uncor}	p_{FDR}	partial R^2	β	SE	t-stat	p_{uncor}	p_{FDR}	partial R^2	p_{FDR}
**Reappraisal**	.010	.024	.438	.665	.944	.007	-.001	.023	-.061	.952	.952	.001	.897
**TIV**	.003	.001	4.475	<.001*	.197	.530	.003	.001	4.368	<.001*	.225	.504	.999
**Age**	.006	.021	.296	.631	.770	.007	-.002	.020	-.123	.511	.712	<.001	.949
**Model**	N = 27, R^2 = .479, F = 7.0, p = .002*						N = 27, R^2 = .472, F= 6.84, p = .002*						

**Left hippocampus**

	E2 condition						PLAC condition						Z test
	β	SE	t-stat	p_{uncor}	p_{FDR}	partial R^2	β	SE	t-stat	p_{uncor}	p_{FDR}	partial R^2	p
**Reappraisal**	<.001	.013	-.066	.948		.001	<-.001	.013	-.023	.982		.001	.976
**TIV**	.002	<.001	4.690	<.001*		.530	.002	>.001	4.364	<.001*		.500	.774
**Age**	.009	.012	.771	.448		.027	.006	.012	.550	.588		.014	.869
**Model**	N = 27, R^2 = .490, F = 7.4, p = .001*						N = 27, R^2 = .457, F = 6.4, p = .003*						

Right hippocampus

	E2 condition						PLAC condition						Z test
	β	SE	t-stat	p_{uncor}	p_{FDR}	partial R^2	β	SE	t-stat	p_{uncor}	p_{FDR}	partial R^2	p
**Reappraisal**	.006	.014	.415	.682		.007	.005	.014	.359	.723		.004	.965
**TIV**	.002	<.001	4.470	<.001*		.522	.001	<.001	4.000	<.001*		.476	.706
**Age**	.007	.012	.556	.584		.016	.003	.012	.271	.789		.005	.837
**Model**	N = 27, R^2 = .474, F = 6.9, p = .002*						N = 27, R^2 = .424, F = 5.6, p = .005*						

Left para-hippocampus

	E2 condition						PLAC condition						Z test
	β	SE	t-stat	p_{uncor}	p_{FDR}	partial R^2	β	SE	t-stat	p_{uncor}	p_{FDR}	partial R^2	p
**Reappraisal**	.001	.001	.095	.925		.002	-.002	.013	-.013	.898		<.001	.875
**TIV**	.001	<.001	2.994	.007*		.328	.001	< .001	3.707	.001*		.407	.789
**Age**	.011	.013	.861	.398		.027	.009	.011	.805	.429		.020	.922
**Model**	N = 27, R^2 = .283, F = 3.0, p = .050						N = 27, R^2 = .374, F = 4.6, p = .012*						

Right para-hippocampus

	E2 condition						PLAC condition						Z test
	β	SE	t-stat	p_{uncor}	p_{FDR}	partial R^2	β	SE	t-stat	p_{uncor}	p_{FDR}	partial R^2	p

**Reappraisal**	.004	.012	.348	.731		.006	-.006	.012	-.496	.625		.016	.552
**TIV**	.001	<.001	3.679	.001*		.442	.001	<.001	3.674	.001*		.405	.964
**Age**	.002	.011	.134	.895		<.001	-.005	.011	-.433	.669		.010	.690
**Model**	N = 27, R^2 = .384, F = 4.7, p = .010*						N = 27, R^2 = .400, F = 5.1, p = .008*						

Left striatum

	E2 condition						PLAC condition						Z test
	β	SE	t-stat	p_{uncor}	p_{FDR}	partial R^2	β	SE	t-stat	p_{uncor}	p_{FDR}	partial R^2	p_{FDR}
**Reappraisal**	-.102	.039	-2.600	.016*	.064	.148	-.081	.036	-2.269	.033*	.132	.097	.897
**TIV**	.006	.001	6.136	<.001*	.012*	.500	.066	.001	6.7224	<.001*	.003*	.526	.999
**Age**	.031	.045	.889	.383	. 692	.002	.035	.032	1.669	.296	.712	<.001	.949
**Model**	N = 27, R^2 = .653, F = 14.4, p < .001*						N = 27, R^2 = .681, F= 16.4, p < .001*						

Right striatum

	E2 condition						PLAC condition						Z test
	β	SE	t-stat	p_{uncor}	p_{FDR}	partial R^2	β	SE	t-stat	p_{uncor}	p_{FDR}	partial R^2	p_{FDR}
**Reappraisal**	-.114	.038	-3.029	.006*	.048*	.213	-.090	.039	-2.353	.028*	.132	.125	.897
**TIV**	.006	.001	6.557	<.001*	.009*	.553	.007	.001	6.743	<.001*	.003*	.582	.999
**Age**	.025	.034	.744	.465	.692	.001	.015	.035	.441	.663	.758	.014	.949
**Model**	N = 27, R^2 = .689, F = 17, p < .001*						N = 27, R^2 = .689, F= 17, p < .001*						

Left ventral striatum

	E2 condition						PLAC condition						Z test
	β	SE	t-stat	p_{uncor}	p_{FDR}	partial R^2	β	SE	t-stat	p_{uncor}	p_{FDR}	partial R^2	p
**Reappraisal**	-.004	.007	-.565	.578		.009	-.007	.006	-1.114	.277		.038	.733
**TIV**	.001	<.001	4.262	<.001*		.477	<.001	< .001	4.516	<.001*		.534	.947

	β	SE	t-stat	p_{uncor}	p_{FDR}	partial R^2	β	SE	t-stat	p_{uncor}	p_{FDR}	partial R^2	p
**Age**	.001	.006	.355	.726		.007	-.001	.006	-.260	.797		.006	.662
**Model**	N = 27, R^2 = .448, F = 6.2, p = .003*						N = 27, R^2 = .499, F = 7.6, p = .001*						

**Right ventral striatum**													
	**E2 condition**						**PLAC condition**						**Z test**
	β	SE	t-stat	p_{uncor}	p_{FDR}	partial R^2	β	SE	t-stat	p_{uncor}	p_{FDR}	partial R^2	p
**Reappraisal**	-.012	.005	-2.400	.025*		.133	-.014	.006	-2.204	.038*		.159	.836
**TIV**	.001	<.001	5.804	<.001*		.567	.001	<.001	4.435	<.001*		.498	.847
**Age**	.003	.005	.585	.564		<.001	.002	.006	-.294	.771		.010	.551
**Model**	N = 27, R^2 = .628, F = 12.9, p < .001*						N = 27, R^2 = .526, F = 8.5, p < .001*						

**Left dorsal striatum**													
	**E2 condition**						**PLAC condition**						**Z test**
	β	SE	t-stat	p_{uncor}	p_{FDR}	partial R^2	β	SE	t-stat	p_{uncor}	p_{FDR}	partial R^2	p
**Reappraisal**	-.092	.035	-2.649	.014*		.163	-.070	.032	-2.223	.036*		.094	.651
**TIV**	.005	.001	5.747	<.001*		.466	.005	> .001	6.320	<.001*		.489	.976
**Age**	.024	.031	.775	.446		.005	.035	.029	1.237	.229		<.001	.792
**Model**	N = 27, R^2 = .630, F = 13.0, p < .001*						N = 27, R^2 = .655, F = 14.6, p < .001*						

**Right dorsal striatum**													
	**E2 condition**						**PLAC condition**						**Z test**
	β	SE	t-stat	p_{uncor}	p_{FDR}	partial R^2	β	SE	t-stat	p_{uncor}	p_{FDR}	partial R^2	p
**Reappraisal**	-.085	.033	-2.607	.016*		.206	-.071	.0323	-2.177	.040*		.112	.766
**TIV**	.005	.001	6.248	<.001*		.430	.008	.001	6.597	<.001*		.567	.791
**Age**	.005	.030	.174	.863		.001	.013	.030	.438	.665		.013	.851

**Model**	N = 27, R^2 = .667, F = 15.4, p < .001*						N = 27, R^2 = .678, F = 16.1, p < .001*						

**Left caudoventral striatum**													
	**E2 condition**						**PLAC condition**						**Z test**
	β	SE	t-stat	p_{uncor}	p_{FDR}	partial R^2	β	SE	t-stat	p_{uncor}	p_{FDR}	partial R^2	p
**Reappraisal**	-.005	.002	-2.731	.012*		.154	-.004	.002	-2.104	.047*		.149	.636
**TIV**	<.001	<.001	5.914	<.001*		.481	<.001	<.001	5.605	<.001*		.567	.772
**Age**	.002	.002	1.291	.208		.001	<.001	.002	-.139	.890		.009	.309
**Model**	N = 27, R^2 = .643, F = 13.8, p < .001*						N = 27, R^2 = .616, F = 12.3, p < .001*						

**Right caudoventral striatum**													
	**E2 condition**						**PLAC condition**						**Z test**
	β	SE	t-stat	p_{uncor}	p_{FDR}	partial R^2	β	SE	t-stat	p_{uncor}	p_{FDR}	partial R^2	p
**Reappraisal**	-.006	.002	3-382	.003*		.279	-.003	.002	-1.121	.274		.045	.207
**TIV**	<.001	<.001	5.041	<.001*		.475	<.001	<.001	4.921	<.001*		.541	.526
**Age**	.001	.002	.866	.396		.003	-.001	.002	-.485	.632		.022	.356
**Model**	N = 27, R^2 = .606, F = 11.8, p < .001*						N = 27, R^2 = .547, F = 9.2, p < .001*						

Rumination

Tab. S6 | Statistical parameters from robust mixed linear regression analysing the relationship between emotion regulation strategy (trait reappraisal and rumination) and regional gray matter volume (GMV) under E2 and placebo (PLAC) conditions. Slope coefficients (β), standard errors (SE), t-statistics, and uncorrected as well as false discovery rate (FDR)-corrected p-values for the association between E2 increase and GMV across both drug conditions are reported. Additionally, p-values from Z-transformed slope comparisons (E2 vs. PLAC) are included to assess condition-specific differences in association strength. Significant associations (p < .05) are marked with an asterisk (*).

**Left amygdala**													
	**E2 condition**						**PLAC condition**						**Z test**
	β	SE	t-stat	p_{uncor}	p_{FDR}	partial R^2	β	SE	t-stat	p_{uncor}	p_{FDR}	partial R^2	p_{FDR}
**Rumination**	.007	.007	1.028	.315	.613	.051	.006	.007	.786	.440	.741	.029	.938
**TIV**	.001	<.001	5.593	<.001*	<.001*	.613	.001	<.001	4.903	<.001*	<.001*	.548	.987
**Age**	.005	.008	.668	.511	.772	.017	.006	.009	.639	.529	.884	.019	.981
**Model**	N = 27, R^2 = .586, F = 10.8, p < .001*						N = 27, R^2 = .518, F= 8.2, p < .001*						

**Right amygdala**													
	**E2 condition**						**PLAC condition**						**Z test**
	β	SE	t-stat	p_{uncor}	p_{FDR}	partial R^2	β	SE	t-stat	p_{uncor}	p_{FDR}	partial R^2	p_{FDR}
**Rumination**	.006	.008	.775	.089	.613	.024	.004	.009	.472	.642	.741	.010	.938
**TIV**	.002	<.001	5.534	<.001*	<.001*	.569	.002	<.001	5.230	<.001*	<.001*	.585	.987
**Age**	.011	.010	1.111	.661	.772	.024	.007	.010	.583	.736	.884	.0134	.981
**Model**	N = 27, R^2 = .575, F = 10.4, p < .001*						N = 27, R^2 = .548, F = 9.3, p < .001*						

**Left ACC**													
	**E2 condition**						**PLAC condition**						**Z test**
	β	SE	t-stat	p_{uncor}	p_{FDR}	partial R^2	β	SE	t-stat	p_{uncor}	p_{FDR}	partial R^2	p_{FDR}
**Rumination**	.017	.030	.568	.576	.619	.015	.014	.029	.463	.648	.741	.012	.938
**TIV**	.006	.001	5.585	<.001*	<.001*	.611	.006	.001	5.784	<.001*	<.001*	.620	.987

**Age**	-.051	.035	-1.457	.159	.772	.092	-.059	.035	-1.699	.103	.822	.120	.981
**Model**	N = 27, R^2 = .631, F = 13.1, p < .001*						N = 27, R^2 = .652, F= 14.4, p < .001*						

**Right ACC**													
	**E2 condition**						**PLAC condition**						**Z test**
	β	SE	t-stat	p_{uncor}	p_{FDR}	partial R^2	β	SE	t-stat	p_{uncor}	p_{FDR}	partial R^2	p_{FDR}
**Rumination**	.014	.028	.504	.619	.619	.013	.009	.027	.327	.747	.747	.013	.938
**TIV**	.006	.001	5.846	<.001*	<.001*	.622	.006	.001	6.430	<.001*	<.001*	.625	.987
**Age**	-.018	.033	-.536	.597	.772	.010	-.025	.031	-.789	.439	.884	.015	.981
**Model**	N = 27, R^2 = .623, F = 12.7, p < .001*						N = 27, R^2 = .67, F = 15.6, p < .001*						

**Left perigenual ACC**													
	**E2 condition**						**PLAC condition**						**Z test**
	β	SE	t-stat	p_{uncor}	p_{FDR}	partial R^2	β	SE	t-stat	p_{uncor}	p_{FDR}	partial R^2	p
**Rumination**	.005	.021	.232	.819		.003	.003	.021	.146	.885		.001	.954
**TIV**	.004	.001	5.536	<.001*		.614	.004	.001	5.550	<.001*		.614	.916
**Age**	-.042	.024	-1.752	.093		.135	-.047	.025	-1.873	.074		.148	.907
**Model**	N = 27, R^2 = .636, F = 13.4, p < .001*						N = 27, R^2 = .642, F = 13.7, p < .001*						

**Right perigenual ACC**													
	**E2 condition**						**PLAC condition**						**Z test**
	β	SE	t-stat	p_{uncor}	p_{FDR}	partial R^2	β	SE	t-stat	p_{uncor}	p_{FDR}	partial R^2	p
**Rumination**	.005	.020	.265	.793		.005	.001	.019	.052	.959		.005	.878
**TIV**	.004	.001	5.64	<.001*		.599	.004	.001	6.072	<.001*		.594	.829
**Age**	-.016	.023	-.698	.492		.017	-.021	.023	-.940	.357		.017	.874
**Model**	N = 27, R^2 = .610, F = 12.0, p < .001*						N = 27, R^2 = .650, F = 14.2, p < .001*						

**Left subgenual ACC**													
	**E2 condition**						**PLAC condition**						**Z test**
	β	SE	t-stat	p_{uncor}	p_{FDR}	partial R^2	β	SE	t-stat	p_{uncor}	p_{FDR}	partial R^2	p
**Rumination**	.012	.009	1.273	.216		.067	.011	.009	1.223	.234		.074	.947
**TIV**	.001	<.001	4.386	<.001*		.476	.001	<.001	4.622	<.001*		.485	.953
**Age**	-.004	.011	-.383	.706		.004	-.009	.011	-.858	.400		.024	.748
**Model**	N = 27, R^2 = .623, F = 10.6, p < .001*						N = 27, R^2 = .546, F = 8.8, p < .001*						

**Right subgenual ACC**													
	**E2 condition**						**PLAC condition**						**Z test**
	β	SE	t-stat	p_{uncor}	p_{FDR}	partial R^2	β	SE	t-stat	p_{uncor}	p_{FDR}	partial R^2	p
**Rumination**	.007	.007	.968	.343		.039	.006	.007	.951	.352		.050	.973
**TIV**	.001	<.001	5.618	<.001*		.612	.001	<.001	5.877	<.001*		.612	.956
**Age**	-.004	.008	-.523	.606		.011	-.008	.008	-1.006	.325		.035	.746
**Model**	N = 27, R^2 = .609, F = 16.9, p < .001*						N = 27, R^2 = .641, F = 13.7, p < .001*						

**Left dorsal ACC**													
	**E2 condition**						**PLAC condition**						**Z test**
	β	SE	t-stat	p_{uncor}	p_{FDR}	partial R^2	β	SE	t-stat	p_{uncor}	p_{FDR}	partial R^2	p
**Rumination**	<.001	.003	.074	.942		.002	-.001	.003	-.244	.809		<.001	.828
**TIV**	<.001	<.001	3.923	<.001*		.431	<.001	<.001	4.514	<.001*		.465	.931
**Age**	-.004	.004	-1.194	.245		.066	-.005	.003	-1.361	.187		.075	.986
**Model**	N = 27, R^2 = .497, F = 7.6, p = .001*						N = 27, R^2 = .535, F = 8.8, p < .001*						

Right dorsal ACC

	E2 condition						PLAC condition						Z test
	β	SE	t-stat	p_{uncor}	p_{FDR}	partial R^2	β	SE	t-stat	p_{uncor}	p_{FDR}	partial R^2	p
**Rumination**	.002	.004	.423	.676		.009	.001	.004	.266	.793		.004	.912
**TIV**	<.001	<.001	3.609	.002*		.393	.001	<.001	3.627	.001*		.394	.981
**Age**	.002	.005	.493	.627		.010	.003	.005	.574	.571		.014	.953
**Model**	N = 27, R^2 = .366, F = 4.4, p = .013*						N = 27, R^2 =.367, F = 4.4, p =.013*						

**Left total hippocampus**													
	E2 condition						PLAC condition						Z test
	β	SE	t-stat	p_{uncor}	p_{FDR}	partial R^2	β	SE	t-stat	p_{uncor}	p_{FDR}	partial R^2	p_{FDR}
**Rumination**	.030	.018	1.644	.114	.455	.125	.025	.016	1.530	.137	.741	.11	.938
**TIV**	.003	.001	4.513	<.001*	<.001*	.517	.003	.001	4.867	<.001*	<.001*	.550	.987
**Age**	.020	.021	.932	.361	.772	.040	.015	.019	.762	.454	.884	.026	.981
**Model**	N = 27, R^2 = .498, F = 7.6, p = .001*						N = 27, R^2 = .653, F= 8.6, p = .001*						

**Right total hippocampus**													
	E2 condition						PLAC condition						Z test
	β	SE	t-stat	p_{uncor}	p_{FDR}	partial R^2	β	SE	t-stat	p_{uncor}	p_{FDR}	partial R^2	p_{FDR}
**Rumination**	.013	.018	.752	.460	.613	.014	.019	.016	1.150	.262	.741	.056	.938
**TIV**	.003	.001	4.498	<.001*	<.001*	.538	.003	.001	4.507	<.001*	<.001*	.521	.987
**Age**	.006	.021	.293	.772	.772	.009	-.003	.019	-.148	.884	.884	<.001	.981
**Model**	N = 27, R^2 = .481, F = 7.1, p = .001*						N = 27, R^2 = .501, F= 7.7, p = .001*						

**Left hippocampus**													
	E2 condition						PLAC condition						Z test
	β	SE	t-stat	p_{uncor}	p_{FDR}	partial R^2	β	SE	t-stat	p_{uncor}	p_{FDR}	partial R^2	p

**Rumination**	.008	.010	.836	.412		.037	.007	.010	.670	.510		.028	.905
**TIV**	.002	<.001	4.820	<.001*		.541	.002	<.001	4.434	<.001*		.509	.770
**Age**	.010	.012	.816	.423		.029	.007	.012	.587	.564		.015	.869
**Model**	N = 27, R^2 = .465, F = 6.7, p = .002*						N = 27, R^2 = .466, F = 6.7, p = .002*						

**Right hippocampus**													
	**E2 condition**						**PLAC condition**						**Z test**
	β	SE	t-stat	p_{uncor}	p_{FDR}	partial R^2	β	SE	t-stat	p_{uncor}	p_{FDR}	partial R^2	p
**Rumination**	.008	.010	.731	.472		.022	.007	.010	.726	.476		.018	.615
**TIV**	.002	<.001	4.575	<.001*		.531	.001	<.001	4.093	<.001*		.484	.899
**Age**	.007	.012	.566	.577		.019	.003	.012	.264	.794		.006	.914
**Model**	N = 27, R^2 = .484, F = 7.2, p = .001*						N = 27, R^2 = .436, F = 5.9, p = .004*						

**Left para-hippocampus**													
	**E2 condition**						**PLAC condition**						**Z test**
	β	SE	t-stat	p_{uncor}	p_{FDR}	partial R^2	β	SE	t-stat	p_{uncor}	p_{FDR}	partial R^2	p
**Rumination**	.022	.010	2.199	.038*		.198	.018	.009	2.082	.049*		.180	.792
**TIV**	.001	<.001	3.599	.002*		.387	.001	<.001	4.228	<.001*		.462	.909
**Age**	.013	.012	1.070	.296		.038	.009	.010	.898	.379		.028	.838
**Model**	N = 27, R^2 = .509, F = 8.0, p < .001*						N = 27, R^2 = .487, F = 7.3, p = .001*						

**Right para-hippocampus**													
	**E2 condition**						**PLAC condition**						**Z test**
	β	SE	t-stat	p_{uncor}	p_{FDR}	partial R^2	β	SE	t-stat	p_{uncor}	p_{FDR}	partial R^2	p
**Rumination**	.006	.009	.624	.539		.018	.012	.009	1.352	.190		.088	.629
**TIV**	.001	<.001	3.733	.001*		.450	.001	<.001	3.716	.001*		.423	.898

**Age**	.002	.022	.183	.856		.001	-.005	.011	-.444	.662		.011	.661
**Model**	N = 27, R^2 = .390, F = 4.9, p = .009*						N = 27, R^2 = .428, F = 5.7, p = .004*						

**Left striatum**													
	**E2 condition**						**PLAC condition**						**Z test**
	β	SE	t-stat	p_{uncor}	p_{FDR}	partial R^2	β	SE	t-stat	p_{uncor}	p_{FDR}	partial R^2	p_{FDR}
**Rumination**	.030	.032	.953	.351	.613	.029	.019	.028	.695	.500	.741	.016	.938
**TIV**	.006	.001	5.763	<.001*	<.001*	.455	.006	.001	6.597	<.001*	<.001*	.497	.987
**Age**	.013	.038	.344	.734	.772	.004	.011	.033	.341	.736	.884	.001	.981
**Model**	N = 27, R^2 = .604, F = 11.7, p < .001*						N = 27, R^2 = .664, F = 15.1, p < .001*						

**Right striatum**													
	**E2 condition**						**PLAC condition**						**Z test**
	β	SE	t-stat	p_{uncor}	p_{FDR}	partial R^2	β	SE	t-stat	p_{uncor}	p_{FDR}	partial R^2	p_{FDR}
**Rumination**	.052	.029	1.811	.083	.455	.040	.022	.031	.717	.481	.741	.012	.938
**TIV**	.007	.001	7.277	<.001*	<.001*	.493	.007	.001	6.453	<.001*	<.001*	.543	.987
**Age**	.024	.034	.702	.490	.772	.002	.010	.037	.262	.795	.884	.017	.981
**Model**	N = 27, R^2 = .714, F = 19.1, p < .001*						N = 27, R^2 = .656, F = 14.6, p < .001*						

**Left ventral striatum**													
	**E2 condition**						**PLAC condition**						**Z test**
	β	SE	t-stat	p_{uncor}	p_{FDR}	partial R^2	β	SE	t-stat	p_{uncor}	p_{FDR}	partial R^2	p
**Rumination**	.005	.005	.947	.354		.031	.004	.005	.874	.391		.040	.912
**TIV**	.001	<.001	4.391	<.001*		.482	.001	<.001	4.777	<.001*		.532	.981
**Age**	.002	.006	.319	.753		.007	-.002	.006	-.371	.714		.007	.627
**Model**	N = 27, R^2 = .472, F = 6.8, p = .002*						N = 27, R^2 = .528, F = 8.6, p < .001*						

**Right ventral striatum**													
	**E2 condition**						**PLAC condition**						**Z test**
	β	SE	t-stat	p_{uncor}	p_{FDR}	partial R^2	β	SE	t-stat	p_{uncor}	p_{FDR}	partial R^2	p
**Rumination**	.005	.004	1.068	.297		.015	-.001	.006	-.097	.924		.002	.466
**TIV**	.001	<.001	5.903	<.001*		.527	.001	<.001	3.740	.001*		.442	.515
**Age**	.005	.005	.944	.355		.001	-.003	.007	-.527	.603		.013	.320
**Model**	N = 27, R^2 = .613, F = 12.1, p < .001*						N = 27, R^2 = .411, F = 5.3, p = .006*						

**Left dorsal striatum**													
	**E2 condition**						**PLAC condition**						**Z test**
	β	SE	t-stat	p_{uncor}	p_{FDR}	partial R^2	β	SE	t-stat	p_{uncor}	p_{FDR}	partial R^2	p
**Rumination**	.024	.029	.822	.420		.029	.017	.025	.685	.500		.012	.856
**TIV**	.005	.001	5.048	<.001*		.418	.005	.001	6.283	<.001*		.459	.820
**Age**	-.003	.034	-.098	.923		.007	.015	.029	.530	.602		.001	.627
**Model**	N = 27, R^2 = .546, F = 9.2, p < .001*						N = 27, R^2 = .640, F = 13.6, p < .001*						

**Right dorsal striatum**													
	**E2 condition**						**PLAC condition**						**Z test**
	β	SE	t-stat	p_{uncor}	p_{FDR}	partial R^2	β	SE	t-stat	p_{uncor}	p_{FDR}	partial R^2	p
**Rumination**	.040	.026	1.527	.140		.038	.017	.027	.648	.523		.012	.541
**TIV**	.006	.001	6.513	<.001*		.471	.006	.001	6.299	<.001*		.532	.930
**Age**	.012	.031	.381	.707		.003	.003	.032	.096	.924		.016	.842
**Model**	N = 27 R^2 = .667, F = 15.3, p < .001*						N = 27, R^2 = .647, F = 14.0, p < .001*						

Left caudoventral striatum

	E2 condition						PLAC condition						Z test
	β	SE	t-stat	p_{uncor}	p_{FDR}	partial R^2	β	SE	t-stat	p_{uncor}	p_{FDR}	partial R^2	p
**Rumination**	<.001	.002	.241	.812		<.001	.001	.002	.494	.626		.014	.857
**TIV**	<.001	.001	5.066	<.001*		.427	<.001	<.001	4.812	<.001*		.520	.744
**Age**	.002	.002	1.066	.298		.002	-.001	.002	-.370	.715		.012	.305
**Model**	N = 27, R^2 = .534, F = 8.8, p < .001*						N = 27, R^2 = .527, F = 8.5, p < .001*						

**Right caudoventral striatum**													
	E2 condition						PLAC condition						Z test
	β	SE	t-stat	p_{uncor}	p_{FDR}	partial R^2	β	SE	t-stat	p_{uncor}	p_{FDR}	partial R^2	p
**Rumination**	.003	.002	1.778	.089		.108	.002	.002	.940	.357		.036	.574
**TIV**	<.001	.001	3.997	.001*		.409	<.001	.001	4.738	<.001*		.535	.533
**Age**	.001	.002	.444	.662		.001	-.001	.002	-.610	.548		.025	.455
**Model**	N = 27, R^2 = .454, F = 6.4, p =.003*						N = 27, R^2 = .533, F = 8.7, p < .001*						

Interaction E2 x reappraisal.

Tab. S7 | Statistical parameters from robust mixed linear regression analysing the relationship between emotion regulation strategy (trait reappraisal and rumination) by E2 increase and regional gray matter volume (GMV) under E2 and placebo (PLAC) conditions. Slope coefficients (β), standard errors (SE), t-statistics, and uncorrected as well as false discovery rate (FDR)-corrected p-values for the association between E2 increase and GMV across both drug conditions are reported. Additionally, p-values from Z-transformed slope comparisons (E2 vs. PLAC) are included to assess condition-specific differences in association strength. Significant associations (p < .05) are marked with an asterisk (*).

**Left amygdala**													
	**E2 condition**						**PLAC condition**						**Z test**
	β	SE	t-stat	p_{uncor}	p_{FDR}	partial R^2	β	SE	t-stat	p_{uncor}	p_{FDR}	partial R^2	p_{FDR}
**ΔE2 x Reappraisal**	-.026	.020	-1.321	.530	.991	.315	-.004	-.009	-.507	.617	.855	.133	.964
**TIV**	.002	<.001	5.297	<.001*	<.001*	.652	.001	<.001	4.921	<.001*	<.001*	.586	.986
**Age**	.005	.011	.470	.821	.932	.004	.001	.011	-.072	.943	.943	<.001	.958
**Model**	N = 21, R^2 = .645, F = 10.3, p < .001*						N = 24, R^2 = .558, F= 8.4, p = .001*						

**Right amygdala**													
	**E2 condition**						**PLAC condition**						**Z test**
	β	SE	t-stat	p_{uncor}	p_{FDR}	partial R^2	β	SE	t-stat	p_{uncor}	p_{FDR}	partial R^2	p_{FDR}
**ΔE2 x Reappraisal**	.002	.023	.092	.928	.991	.285	-.003	.009	-.335	.741	.855	.171	.964
**TIV**	.002	<.001	5.228	<.001*	<.001*	.628	.002	<.001	5.545	<.001*	<.001*	.640	.986
**Age**	-.002	.023	-.087	.932	.932	.006	-.004	.011	-.313	.758	.518	.002	.958
**Model**	N = 21, R^2 = .640, F = 10.1, p < .001*						N = 24, R^2 = .622, F = 11.0, p < .001*						

**Left ACC**													
	**E2 condition**						**PLAC condition**						**Z test**
	β	SE	t-stat	p_{uncor}	p_{FDR}	partial R^2	β	SE	t-stat	p_{uncor}	p_{FDR}	partial R^2	p_{FDR}

**ΔE2 x Reappraisal**	-.084	.072	-1.176	.256	.991	.437	-.012	.030	-.402	.692	.855	.308	.964
**TIV**	.007	.001	6.785	<.001*	<.001*	.747	.006	.001	6.708	<.001*	<.001*	.719	.986
**Age**	-.087	.038	-2.308	.034*	.271	.243	-.073	.038	-1.954	.065	.943	.182	.958
**Model**	N = 21, R^2 = .784, F =20.5, p < .001*						N = 24, R^2 = .736, F= 18.6, p < .001*						

**Right ACC**													
	**E2 condition**						**PLAC condition**						**Z test**
	β	SE	t-stat	p_{uncor}	p_{FDR}	partial R^2	β	SE	t-stat	p_{uncor}	p_{FDR}	partial R^2	p_{FDR}
**ΔE2 x Reappraisal**	-.027	.075	-.362	.722	.991	.274	-.005	.025	-.213	.834	.855	.464	.964
**TIV**	.006	.001	6.107	<.001*	<.001*	.691	.006	.001	7.764	<.001*	<.001*	.776	.986
**Age**	-.038	.039	-.975	.343	.932	.044	-.034	.032	-1.06	.302	.943	.062	.958
**Model**	N = 21, R^2 = .721, F = 14.6, p < .001*						N = 24, R^2 = .772, F = 22.6, p < .001*						

**Left perigenual ACC**													
	**E2 condition**						**PLAC condition**						**Z test**
	β	SE	t-stat	p_{uncor}	p_{FDR}	partial R^2	β	SE	t-stat	p_{uncor}	p_{FDR}	partial R^2	p
**ΔE2 x Reappraisal**	-.076	.048	-1.585	.131		.463	-.016	.022	-.711	.486		.216	.256
**TIV**	.005	.001	6.944	<.001*		.764	.004	.001	6.094	<.001*		.687	.704
**Age**	-.065	.025	-2.579	.020*		.304	-.058	.028	-2.060	.053		.197	.856
**Model**	N = 21, R^2 = .795, F = 4.4, p < .001*						N = 24, R^2 = .703, F = 15.8, p < .001*						

**Right perigenual ACC**													
	**E2 condition**						**PLAC condition**						**Z test**

	β	SE	t-stat	p_{uncor}	p_{FDR}	partial R^2	β	SE	t-stat	p_{uncor}	p_{FDR}	partial R^2	p
**ΔE2 x Reappraisal**	-.018	.054	-.328	.747		.235	-.005	.019	-.261	.797		.446	.822
**TIV**	.004	.001	5.567	<.001*		.649	.004	<.001	7.082	<.001*		.750	.953
**Age**	-.029	.028	-1.022	.321		.046				.254		.071	.969
**Model**	N = 21, R^2 = .686, F =12.4, p < .001*						N = 24, R^2 = .741, F = 19.1, p < .001*						
**Left subgenual ACC**													
	**E2 condition**						**PLAC condition**						**Z test**
	β	SE	t-stat	p_{uncor}	p_{FDR}	partial R^2	β	SE	t-stat	p_{uncor}	p_{FDR}	partial R^2	p
**ΔE2 x Reappraisal**	-.009	.025	-.363	.721		.241	.004	.009	.424	.677		.379	.628
**TIV**	.002	<.001	4.707	<.001*		.542	.002	.001	5.197	<.001*		.614	.692
**Age**	-.015	.013	-1.109	.283		.048	-.011	.012	-.952	.353		.046	.827
**Model**	N = 21, R^2 = .619, F = 9.2, p = .001*						N = 24, R^2 = .621, F = 10.9, p < .001*						
**Right subgenual ACC**													
	**E2 condition**						**PLAC condition**						**Z test**
	β	SE	t-stat	p_{uncor}	p_{FDR}	partial R^2	β	SE	t-stat	p_{uncor}	p_{FDR}	partial R^2	p
**ΔE2 x Reappraisal**	-.009	.025	-.363	.950		.264	.004	.009	.424	.624		.379	.628
**TIV**	.002	<.001	4.707	<.001*		.688	.002	.001	5.197	<.001*		.723	.692
**Age**	-.015	.013	-1.109	.441		.036	-.011	.012	-.952	.341		.048	.827
**Model**	N = 21, R^2 = .619, F = 9.2, p = .001*						N = 24, R^2 = .621, F = 10.9, p < .001*						
**Left dorsal ACC**													

	E2 condition						PLAC condition						Z test
	β	SE	t-stat	p_{uncor}	p_{FDR}	partial R^2	β	SE	t-stat	p_{uncor}	p_{FDR}	partial R^2	p
**ΔE2 x Reappraisal**	-.001	.020	-.063	.842		.334	.004	.007	.498	.757		.316	.827
**TIV**	.002	<.001	5.664	<.001*		.511	.001	<.001	6.623	<.001*		.588	.608
**Age**	-.008	.011	-.789	.090		.136	-.008	.009	-.976	.156		.117	1
**Model**	N = 21, R^2 = .690, F = 12.6, p < .001*						N = 24, R^2 = .721, F = 17.3, p < .001*						

Right dorsal ACC

	E2 condition						PLAC condition						Z test
	β	SE	t-stat	p_{uncor}	p_{FDR}	partial R^2	β	SE	t-stat	p_{uncor}	p_{FDR}	partial R^2	p
**ΔE2 x Reappraisal**	-.003	.010	-.272	.842		.383	-.003	.004	-.639	.530		.184	.995
**TIV**	.001	<.001	4.093	.002*		.543	<.001	<.001	4.085	.001*		.476	.848
**Age**	-.002	.005	-.275	.842		.004	<.001	.005	.143	.887		.001	.767
**Model**	N = 21, R^2 = .521, F = 6.2, p = .005*						N = 24, R^2 =.460, F = 5.7, p =.006*						

Left total hippocampus

	E2 condition						PLAC condition						Z test
	β	SE	t-stat	p_{uncor}	p_{FDR}	partial R^2	β	SE	t-stat	p_{uncor}	p_{FDR}	partial R^2	p_{FDR}
**ΔE2 x Reappraisal**	.007	.054	.128	.892	.991	.254	-.024	.019	-1.246	.227	.855	.173	.964
**TIV**	.002	.001	3.961	.001*	.001*	.528	.003	.001	4.861	<.001*	<.001*	.586	.986
**Age**	-.007	.028	-.244	.810	.932	.003	-.003	.024	-.114	.910	.943	.001	.958
**Model**	N = 21, R^2 = .513, F = 6.0, p = .006*						N = 24, R^2 = .552, F= 8.2, p = .001*						

Right total hippocampus													
	**E2 condition**						**PLAC condition**						**Z test**
	β	SE	t-stat	p_{uncor}	p_{FDR}	partial R²	β	SE	t-stat	p_{uncor}	p_{FDR}	partial R²	p_{FDR}
**ΔE2 x Reappraisal**	-.032	.055	-.580	.570	.991	.172	-.018	.019	-.946	.356	.855	.163	.964
**TIV**	.003	.001	4.074	.001*	.001*	.550	.003	.001	4.724	<.001*	<.001*	.577	.986
**Age**	-.017	.028	-.589	.564	.932	.019	-.012	.024	-.484	.634	.943	.007	.958
**Model**	N = 21, R² = .528, F = 6.3, p = .004*						N = 24, R² = .546, F= 8.0, p = .001*						

Left hippocampus													
	**E2 condition**						**PLAC condition**						**Z test**
	β	SE	t-stat	p_{uncor}	p_{FDR}	partial R²	β	SE	t-stat	p_{uncor}	p_{FDR}	partial R²	p
**ΔE2 x Reappraisal**	.004	.026	.158	.876		.315	-.009	.010	-.821	.421		.181	.654
**TIV**	.002	<.001	4.956	<.001*		.612	.002	<.001	4.766	<.001*		.552	.588
**Age**	-.003	.014	-.244	.810		.002	-.008	.013	-.644	.527		.017	.791
**Model**	N = 21, R² = .620, F = 9.3, p = .001*						N = 24, R² = .554, F = 8.3, p = .001*						

Right hippocampus													
	**E2 condition**						**PLAC condition**						**Z test**
	β	SE	t-stat	p_{uncor}	p_{FDR}	partial R²	β	SE	t-stat	p_{uncor}	p_{FDR}	partial R²	p
**ΔE2 x Reappraisal**	-.014	.032	-.417	.682		.129	-.009	.012	-.737	.470		.046	.900
**TIV**	.002	<.001	4.126	.001*		.547	.002	<.001	4.077	.001*		.498	.648
**Age**	<-.001	.017	-.017	.986		<.001	<.001	.016	-.003	.998		<.001	.991

**Model**	N = 21, R^2 = .515, F = 6.0, p = .006*						N = 24, R^2 = .461, F = 5.7, p = .006*						

**Left para-hippocampus**													
	**E2 condition**						**PLAC condition**						**Z test**
	β	SE	t-stat	p_{uncor}	p_{FDR}	partial R^2	β	SE	t-stat	p_{uncor}	p_{FDR}	partial R^2	p
**ΔE2 x Reappraisal**	.003	.032	.086	.933		.184	-.016	.010	-1.618	.121		.191	.575
**TIV**	.001	<.001	2.601	.019*		.333	.001	<.001	4.510	<.001*		.505	.648
**Age**	-.003	.017	-.149	.883		.003	.012	.013	.842	.410		.008	.533
**Model**	N = 21, R^2 = .312, F = 2.6, p = 089						N = 24, R^2 = .514, F = 7.0, p = .002*						

**Right para-hippocampus**													
	**E2 condition**						**PLAC condition**						**Z test**
	β	SE	t-stat	p_{uncor}	p_{FDR}	partial R^2	β	SE	t-stat	p_{uncor}	p_{FDR}	partial R^2	p
**ΔE2 x Reappraisal**	-.020	.026	-.784	.444		.244	-.007	.009	-1.002	.319		.316	.700
**TIV**	.001	<.001	3.528	.003*		.462	.001	<.001	4.505	<.001*		.547	.885
**Age**	-.015	.014	-1.137	.271		.071	-.009	.012	-.717	.482		.033	.704
**Model**	N = 21, R^2 = .489, F = 5.4, p = .008*						N = 24, R^2 = .529, F = 7.5, p = .002*						

**Left striatum**													
	**E2 condition**						**PLAC condition**						**Z test**
	β	SE	t-stat	p_{uncor}	p_{FDR}	partial R^2	β	SE	t-stat	p_{uncor}	p_{FDR}	partial R^2	p_{FDR}
**ΔE2 x Reappraisal**	.001	.085	.011	.991	.991	.450	-009	.028	-.339	.739	.855	.173	.964
**TIV**	.006	.001	4.960	<.001*	<.001*	.520	.007	.001	7.389	<.001*	<.001*	.586	.986

**Age**	-.013	.045	-.280	.783	.932	.077	-.011	.035	-.306	.763	.943	.001	.958
**Model**	N = 21, R^2 = .622, F = 9.3, p =.001 *						N = 24, R^2 = .747, F = 19.7, p < .001*						

**Right striatum**													
	**E2 condition**						**PLAC condition**						**Z test**
	β	SE	t-stat	p_{uncor}	p_{FDR}	partial R^2	β	SE	t-stat	p_{uncor}	p_{FDR}	partial R^2	p_{FDR}
**ΔE2 x Reappraisal**	.034	.094	.366	.719	.991	.425	.006	.031	.185	.855	.855	.402	.964
**TIV**	.006	.001	4.736	<.001*	<.001*	.568	.006	.001	6.934	<.001*	<.001*	.622	.986
**Age**	-.027	.049	-.546	.592	.932	.051	-.037	.039	-.942	.358	.943	.150	.958
**Model**	N = 21, R^2 = .615, F = 9.04, p = .001*						N = 24, R^2 = .737., F = 18.7, p < .001*						

**Left ventral striatum**													
	**E2 condition**						**PLAC condition**						**Z test**
	β	SE	t-stat	p_{uncor}	p_{FDR}	partial R^2	β	SE	t-stat	p_{uncor}	p_{FDR}	partial R^2	p
**ΔE2 x Reappraisal**	.001	.014	.079	.938		.259	.007	.005	1.424	.170		.334	.688
**TIV**	.001*	<.001	2.879	.010*		.497	.001	<.001	4.951	<.001*		.607	.399
**Age**	-.009	.007	-1.257	.226		.024	-.005	.006	-.758	.457		.043	.643
**Model**	N = 21, R^2 = .431, F = 4.3, p = .020*						N = 24, R^2 = .625, F = 11.1, p < .001*						

**Right ventral striatum**													
	**E2 condition**						**PLAC condition**						**Z test**
	β	SE	t-stat	p_{uncor}	p_{FDR}	partial R^2	β	SE	t-stat	p_{uncor}	p_{FDR}	partial R^2	p
**ΔE2 x Reappraisal**	-.003	.013	-.242	.811		.275	.010	.005	1.830	.082		.355	.340

**TIV**	.001	<.001	4.903	<.001*		.600	.001	<.001	4.227	<.001*		.524	.456
**Age**	-.001	.007	-.143	.888		.020	-.009	.007	-1.380	.183		.122	.387
**Model**	N = 21, R^2 = .607, F = 8.7, p = .001*						N = 24, R^2 = .603, F = 10.1, p < .001*						

**Left dorsal striatum**													
	**E2 condition**						**PLAC condition**						**Z test**
	β	SE	t-stat	p_{uncor}	p_{FDR}	partial R^2	β	SE	t-stat	p_{uncor}	p_{FDR}	partial R^2	p
**ΔE2 x Reappraisal**	.011	.075	.143	.888		.466	-.017	.024	-.717	.482		.416	.724
**TIV**	.005	.001	4.480	<.001*		.483	.006	.001	7.210	<.001*		.596	.579
**Age**	-.018	.040	-.443	.664		.081	-.013	.030	-.418	.681		.160	.922
**Model**	N = 21, R^2 = .582, F = 7.8, p = .002*						N = 24, R^2 = .738, F = 18.4, p < .001*						

**Right dorsal striatum**													
	**E2 condition**						**PLAC condition**						**Z test**
	β	SE	t-stat	p_{uncor}	p_{FDR}	partial R^2	β	SE	t-stat	p_{uncor}	p_{FDR}	partial R^2	p
**ΔE2 x Reappraisal**	.031	.081	.386	.704		.452	-.004	.026	-.146	.886		.417	.680
**TIV**	.005	.001	4.482	<.001*		.553	.006	.001	6.759	<.001*		.653	.727
**Age**	-.028	.043	-.653	.522		.058	-.037	.033	-1.109	.280		.237	.870
**Model**	N = 21 R^2 = .594, F = 8.3, p = .001*						N = 24, R^2 = .727, F = 17.5, p < .001*						

**Left caudoventral striatum**													
	**E2 condition**						**PLAC condition**						**Z test**
	β	SE	t-stat	p_{uncor}	p_{FDR}	partial R^2	β	SE	t-stat	p_{uncor}	p_{FDR}	partial R^2	p

**ΔE2 x Reappraisal**	-.002	.005	-.402	.693		.256	<-.001	.002	-.137	.893		.194	.741
**TIV**	<.001	<.001	4.489	<.001*		.465	<.001	<.001	4.728	<.001*		.556	.671
**Age**	.001	.003	.225	.825		.021	-.001	.002	-.674	.508		.043	.538
**Model**	N = 21, R^2 = .554, F = 7.0, p = .003*						N = 24, R^2 = .558, F = 8.4, p = .001*						

**Right caudoventral striatum**													
	**E2 condition**						**PLAC condition**						**Z test**
	β	SE	t-stat	p_{uncor}	p_{FDR}	partial R^2	β	SE	t-stat	p_{uncor}	p_{FDR}	partial R^2	p
**ΔE2 x Reappraisal**	.006	.005	1.249	.229		.213	-.001	.002	-.453	.655		.084	.184
**TIV**	<.001	<.001	2.777	.013*		.340	<.001	<.001	4.664	<.001*		.556	.340
**Age**	<-.001	.003	-.114	.911		.001	-.002	.003	-.935	.361		.053	.581
**Model**	N = 21, R^2 = .407, F = 3.9, p =.028*						N = 24, R^2 = .556, F = 8.3, p < .001*						

Interaction E2 x rumination.

Tab. S8 | Statistical parameters from robust mixed linear regression analysing the relationship between emotion regulation strategy (trait reappraisal and rumination) by E2 increase and regional gray matter volume (GMV) under E2 and placebo (PLAC) conditions. Slope coefficients (β), standard errors (SE), t-statistics, and uncorrected as well as false discovery rate (FDR)-corrected p-values for the association between E2 increase and GMV across both drug conditions are reported. Additionally, p-values from Z-transformed slope comparisons (E2 vs. PLAC) are included to assess condition-specific differences in association strength. Significant associations (p < .05) are marked with an asterisk (*).

**Left amygdala**													
	**E2 condition**						**PLAC condition**						**Z test**
	β	SE	t-stat	p_{uncor}	p_{FDR}	partial R^2	β	SE	t-stat	p_{uncor}	p_{FDR}	partial R^2	p_{FDR}
**ΔE2 x Rumination**	-.026	.020	-1.321	.204	.896	.364	-.005	.006	-.877	.391	.875	.157	.969
**TIV**	.002	<.001	5.297	<.001*	<.001*	.653	.001	<.001	4.964	<.001*	<.001*	.591	.986
**Age**	.005	.011	.470	.644	.891	.007	-.002	.011	-.153	.880	.884	.001	.958
**Model**	N = 21, R^2 = .680, F = 12.0, p = .001*						N = 24, R^2 = .497, F= 6.6, p < .003*						

**Right amygdala**													
	**E2 condition**						**PLAC condition**						**Z test**
	β	SE	t-stat	p_{uncor}	p_{FDR}	partial R^2	β	SE	t-stat	p_{uncor}	p_{FDR}	partial R^2	p_{FDR}
**ΔE2 x Rumination**	-.025	.024	-1.04	.313	.896	.302	-.004	.006	-.600	.555	.875	.304	.969
**TIV**	.002	<.001	5.335	<.001*	<.001*	.577	.002	<.001	5.46	<.001*	<.001*	.687	.986
**Age**	.010	.013	.762	.457	.891	<.001	-.004	.011	-.365	.719	.595	.171	.958
**Model**	N = 21, R^2 = .686, F = 12.3, p < .001*						N = 24, R^2 = .626, F = 11.2, p < .001*						

**Left ACC**													
	**E2 condition**						**PLAC condition**						**Z test**
	β	SE	t-stat	p_{uncor}	p_{FDR}	partial R^2	β	SE	t-stat	p_{uncor}	p_{FDR}	partial R^2	p_{FDR}

**ΔE2 x Rumination**	.080	.081	.990	.336	.896	.421	.006	.020	.269	.790	.875	.157	.969
**TIV**	.006	.001	4.722	<.001*	<.001*	.582	.006	.001	6.177	<.001*	<.001*	.591	.986
**Age**	-.103	.044	-2.360	.031*	.244	.251	-.071	.038	-1.883	.074	.595	.001	.958
**Model**	N = 21, R^2 = .779, F = 20, p < .001*						N = 24, R^2 = .733, F= 18.3, p < .001*						

**Right ACC**													
	**E2 condition**						**PLAC condition**						**Z test**
	β	SE	t-stat	p_{uncor}	p_{FDR}	partial R^2	β	SE	t-stat	p_{uncor}	p_{FDR}	partial R^2	p_{FDR}
**ΔE2 x Rumination**	.006	.083	.072	.944	.996	.266	.009	.0171	.542	.594	.790	.471	.969
**TIV**	.006	.001	4.885	<.001*	<.001*	.576	.006	.001	7.183	<.001*	<.001*	.749	.986
**Age**	-.038	.045	-.841	.412	.891	.036	-.032	.032	-.990	.334	.884	.053	.958
**Model**	N = 21, R^2 = .721, F = 14.7, p < .001*						N = 24, R^2 = .774, F = 22.8, p < .001*						

**Left perigenual ACC**													
	**E2 condition**						**PLAC condition**						**Z test**
	β	SE	t-stat	p_{uncor}	p_{FDR}	partial R^2	β	SE	t-stat	p_{uncor}	p_{FDR}	partial R^2	p
**ΔE2 x Rumination**	.066	.058	1.134	.273		.420	.001	.015	.066	.949		.193	.281
**TIV**	.004	.001	4.331	<.001*		.586	.004	.001	5.572	<.001*		.648	.785
**Age**	-.079	.031	-2.505	.023*		.293	-.057	.029	-1.964	.064		.182	.605
**Model**	N = 21, R^2 = .765, F = 18.4, p < .001*						N = 24, R^2 = .694, F = 15.1, p < .001*						

**Right perigenual ACC**			
	**E2 condition**	**PLAC condition**	**Z test**

	β	SE	t-stat	p_{uncor}	p_{FDR}	partial R²	β	SE	t-stat	p_{uncor}	p_{FDR}	partial R²	p
**ΔE2 x Rumination**	.008	.060	.131	.897		.228	.008	.013	.601	.555		.452	.995
**TIV**	004	.001	4.406	<.001*		.523	.004	.001	6.486	<.001*		.719	.909
**Age**	-.030	.033	-.925	.368		.042	-.026	.023	-1.119	.276		.060	.922
**Model**	N = 21, R² = .686, F = 12.4, p < .001*						N = 24, R² = .743, F = 19.3, p < .001*						

**Left subgenual ACC**													
	**E2 condition**						**PLAC condition**						**Z test**
	β	SE	t-stat	p_{uncor}	p_{FDR}	partial R²	β	SE	t-stat	p_{uncor}	p_{FDR}	partial R²	p
**ΔE2 x Rumination**	.022	.028	.793	.439		.265	.005	.006	.779	.445		.390	.546
**TIV**	.002	4.287	3.480	.003*		.360	.001	<.001	4.790	<.001*		.577	.934
**Age**	-.020	.015	-1.333	.200		.079	-.011	.012	-.916	.370		.041	.619
**Model**	N = 21, R² = .641, F = 10.1, p < .001*						N = 24, R² = .629, F = 11.3, p < .001*						

**Right subgenual ACC**													
	**E2 condition**						**PLAC condition**						**Z test**
	β	SE	t-stat	p_{uncor}	p_{FDR}	partial R²	β	SE	t-stat	p_{uncor}	p_{FDR}	partial R²	p
**ΔE2 x Rumination**	-.007	.022	-.293	.773		.236	.003	.005	.656	.5119		.386	.675
**TIV**	.002	<.001	4.870	<.001*		.526	.001	<.001	6.222	<.001*		.628	.499
**Age**	-.007	.012	-.572	.575		.024	-.008	.009	-.932	.363		.083	.940
**Model**	N = 21, R² = .700, F = 13.3, p < .001*						N = 24, R² = .726, F = 17.7, p < .001*						

**Left dorsal ACC**

	E2 condition						PLAC condition						Z test
	β	SE	t-stat	p_{uncor}	p_{FDR}	partial R^2	β	SE	t-stat	p_{uncor}	p_{FDR}	partial R^2	p
**ΔE2 x Rumination**	-.004	.009	-.465	.648		.339	<.001	.002	.136	.894		.312	.629
**TIV**	.001	<.001	3.827	.001*		.439	<.001	<.001	4.869	<.001*		.562	.803
**Age**	-.006	.005	-1.330	.201		.086	-.005	.004	-1.478	.155		.117	.897
**Model**	N = 21, R^2 = .630, F = 9.6, p = .001*						N = 24, R^2 = 628, F = 11.3, p < .001*						

Right dorsal ACC

	E2 condition						PLAC condition						Z test
	β	SE	t-stat	p_{uncor}	p_{FDR}	partial R^2	β	SE	t-stat	p_{uncor}	p_{FDR}	partial R^2	p
**ΔE2 x Rumination**	.002	.011	.179	.860		.380	<-.001	.003	-.179	.860		.165	.827
**TIV**	<.001	<.001	3.203	.005*		.422	<.001	<.001	3.781	.001*		.442	.931
**Age**	-.002	.006	-.300	.768		.004	.001	.006	.184	.856		.001	.729
**Model**	N = 21, R^2 = .523, F = 6.2, p = .005*						N = 24, R^2 = .446, F = 5.3, p =.007*						

Left total hippocampus

	E2 condition						PLAC condition						Z test
	β	SE	t-stat	p_{uncor}	p_{FDR}	partial R^2	β	SE	t-stat	p_{uncor}	p_{FDR}	partial R^2	p_{FDR}
**ΔE2 x Rumination**	-.022	.060	-.361	.722	.996	.262	-.013	.013	-1.019	.320	.790	.150	.969
**TIV**	.003	.002	3.473	.003*	.003*	.465	.003	.006	4.797	<.001*	<.001*	.572	.986
**Age**	-.001	.032	-.031	.976	.976	<.001	-.004	.024	-.148	.884	.884	.001	.958
**Model**	N = 21, R^2 = .517, F = 6.08, p = .005*						N = 24, R^2 = .549, F= 8.1, p = .001*						

Right total hippocampus													
	E2 condition						PLAC condition						Z test
	β	SE	t-stat	p_{uncor}	p_{FDR}	partial R^2	β	SE	t-stat	p_{uncor}	p_{FDR}	partial R^2	p_{FDR}
**ΔE2 x Rumination**	-.005	.061	-.083	.935	.996	.147	-.011	.013	-.837	.412	.790	.158	.969
**TIV**	.003	.001	3.271	.005*	.005*	.415	.003	.001	4.644	<.001*	<.001*	.568	.986
**Age**	-.015	.033	-.436	.668	.891	.013	-.011	.025	-.458	.652	.884	.008	.958
**Model**	N = 21, R^2 = .523, F = 6.2, p = .005*						N = 24, R^2 = .543, F= 7.9, p = .001*						

Left hippocampus													
	E2 condition						PLAC condition						Z test
	β	SE	t-stat	p_{uncor}	p_{FDR}	partial R^2	β	SE	t-stat	p_{uncor}	p_{FDR}	partial R^2	p
**ΔE2 x Rumination**	-.015	.029	-.516	.612		.327	-.005	.007	-.631	.535		.168	.725
**TIV**	.002	<.001	4.386	<.001*		.556	.002	<.001	4.562	<.001*		.532	.498
**Age**	.001	.016	.066	.948		.001	-.009	.013	-.672	.510		.017	.629
**Model**	N = 21, R^2 = .625, F = 9.5, p = .001*						N = 24, R^2 = .546, F = 8.0, p = .001*						

Right hippocampus													
	E2 condition						PLAC condition						Z test
	β	SE	t-stat	p_{uncor}	p_{FDR}	partial R^2	β	SE	t-stat	p_{uncor}	p_{FDR}	partial R^2	p
**ΔE2 x Rumination**	-.003	.037	-.075	.941		.114	-.006	.009	-.726	.477		.063	.929
**TIV**	.002	.001	3.273	.005*		.375	.002	<.001	3.983	.001*		.418	.762
**Age**	.001	.020	.050	.961		<.001	<-.001	.016	-.027	.979		.001	.956
**Model**	N = 21, R^2 = .502, F = 5.7, p = .007*						N = 24, R^2 = .457, F = 5.6, p = .006*						

**Left para-hippocampus**													
	**E2 condition**						**PLAC condition**						**Z test**
	β	SE	t-stat	p_{uncor}	p_{FDR}	partial R^2	β	SE	t-stat	p_{uncor}	p_{FDR}	partial R^2	p
**ΔE2 x Rumination**	-.009	.035	-.250	.801		.186	-.009	.007	-1.297	.210		.171	.991
**TIV**	.001	.001	2.298	.035*		.276	.002	<.001	4.395	<.001*		.499	.665
**Age**	<-.001	.019	-.009	.993		<.001	.011	.013	.853	.404		.006	.620
**Model**	N = 21, R^2 = .318, F = 2.6, p = .083						N = 24, R^2 = .493, F = 6.5, p = .003*						

**Right para-hippocampus**													
	**E2 condition**						**PLAC condition**						**Z test**
	β	SE	t-stat	p_{uncor}	p_{FDR}	partial R^2	β	SE	t-stat	p_{uncor}	p_{FDR}	partial R^2	p
**ΔE2 x Rumination**	.003	.030	.099	.922		.213	-.005	.007	-.828	.418		.302	.785
**TIV**	.001	<.001	2.592	.019*		.324	.001	<.001	4.383	<.001*		.532	.732
**Age**	-.015	.016	-.928	.367		.049	-.009	.012	-.703	.490		.034	.749
**Model**	N = 21, R^2 = .460, F = 4.8, p = .013*						N = 24, R^2 = .521, F = 7.3, p = .002*						

**Left striatum**													
	**E2 condition**						**PLAC condition**						**Z test**
	β	SE	t-stat	p_{uncor}	p_{FDR}	partial R^2	β	SE	t-stat	p_{uncor}	p_{FDR}	partial R^2	p_{FDR}
**ΔE2 x Rumination**	<.001	.094	.005	.996	.996	.459	-.008	.019	-.413	.684	.790	.406	.969
**TIV**	.006	.001	4.105	.001*	.001*	.377	.007	.001	7.059	<.001*	<.001*	.610	.986
**Age**	-.009	.051	-.181	.859	.976	.096	-.013	.036	-.351	.729	.884	.155	.958

**Model**	N = 21, R^2 = .629, F = 9.6, p = .001*						N = 24, R^2 = .744, F = 19.4, p < .001*						

**Right striatum**													
	**E2 condition**						**PLAC condition**						**Z test**
	β	SE	t-stat	p_{uncor}	p_{FDR}	partial R^2	β	SE	t-stat	p_{uncor}	p_{FDR}	partial R^2	p_{FDR}
**ΔE2 x Rumination**	-.016	.105	-.150	.883	.996	.421	-.006	.021	-.304	.765	.790	.397	.969
**TIV**	.006	.002	3.930	.001*	.001*	.467	.006	.001	6.327	<.001*	<.001*	.633	.986
**Age**	-.028	.057	-.490	.631	.891	.044	-.053	.038	-1.372	.185	.741	.225	.958
**Model**	N = 21, R^2 = .709, F = 8.8, p = .001*						N = 24, R^2 = .721, F = 17.3, p < .001*						

**Left ventral striatum**													
	**E2 condition**						**PLAC condition**						**Z test**
	β	SE	t-stat	p_{uncor}	p_{FDR}	partial R^2	β	SE	t-stat	p_{uncor}	p_{FDR}	partial R^2	p
**ΔE2 x Rumination**	.007	.016	.455	.655		.296	.005	.003	1.603	.125		.349	.890
**TIV**	.001	<.001	2.507	.023*		.307	.001	<.001	4.631	<.001*		.556	.761
**Age**	-.008	.009	-.955	.353		.061	-.004	.006	-.632	.535		.036	.662
**Model**	N = 21, R^2 = .472, F = 5.1, p = .011*						N = 24, R^2 = .647, F = 12.2, p < .001*						

**Right ventral striatum**													
	**E2 condition**						**PLAC condition**						**Z test**
	β	SE	t-stat	p_{uncor}	p_{FDR}	partial R^2	β	SE	t-stat	p_{uncor}	p_{FDR}	partial R^2	p
**ΔE2 x Rumination**	-.005	.014	-.359	.724		.268	.006	.004	1.530	.142		.290	.461
**TIV**	.001	<.001	4.424	<.001*		.397	.001	<.001	3.810	.001*		.347	.338

**Age**	.003	.008	.322	.751		.026	-.009	.007	-1.281	.215		.175	.271
**Model**	N = 21, R^2 = .633, F = 9.6, p = .001*						N = 24, R^2 = .595, F = 9.8, p < .001*						

**Left dorsal striatum**													
	**E2 condition**						**PLAC condition**						**Z test**
	β	SE	t-stat	p_{uncor}	p_{FDR}	partial R^2	β	SE	t-stat	p_{uncor}	p_{FDR}	partial R^2	p
**ΔE2 x Rumination**	.015	.085	.176	.862		.502	-.012	.016	-.797	.488		.460	.759
**TIV**	.005	.001	3.408	.003*		.244	.006	.001	6.896	<.001*		.505	.495
**Age**	-.032	.046	-.705	.491		.126	-.014	.031	-.449	.658		.224	.736
**Model**	N = 21, R^2 = .572, F = 7.5, p = .002*						N = 24, R^2 = .732, F = 17.9, p < .001*						

**Right dorsal striatum**													
	**E2 condition**						**PLAC condition**						**Z test**
	β	SE	t-stat	p_{uncor}	p_{FDR}	partial R^2	β	SE	t-stat	p_{uncor}	p_{FDR}	partial R^2	p
**ΔE2 x Rumination**	-.005	.091	-.056	.956		.485	-.014	.017	-.808	.428		.485	.925
**TIV**	.005	.001	3.677	.002*		.346	.005	.001	6.098	<.001*		.558	.957
**Age**	-.030	.049	-.611	.549		.081	-.056	.032	-1.758	.094		.351	.659
**Model**	N = 21, R^2 = .588, F = 8.1, p = .002*						N =24, R^2 = .707, F = 15.8, p < .001*						

**Left caudoventral striatum**													
	**E2 condition**						**PLAC condition**						**Z test**
	β	SE	t-stat	p_{uncor}	p_{FDR}	partial R^2	β	SE	t-stat	p_{uncor}	p_{FDR}	partial R^2	p
**ΔE2 x Rumination**	-.003	.005	-.524	.607		.259	-.001	.001	-1.190	.248		.251	.789

**TIV**	<.001	<.001	3.672	.002*		.390	<.001	<.001	5.145	<.001*		.589	.838
**Age**	<.001	.003	.173	.865		.006	-.002	.002	-.788	.440		.069	.546
**Model**	N = 21, R^2 = .531, F = 6.4, p = .004*						N = 24, R^2 = .595, F = 9.8, p < .001*						

**Right caudoventral striatum**													
	**E2 condition**						**PLAC condition**						**Z test**
	β	SE	t-stat	p_{uncor}	p_{FDR}	partial R^2	β	SE	t-stat	p_{uncor}	p_{FDR}	partial R^2	p
**ΔE2 x Rumination**	-.007	.006	-1.283	.217		.206	-.002	.001	-1.331	.198		.157	.336
**TIV**	<.001	<.001	3.182	.006*		.391	<.001	<.001	5.040	<.001*		.590	.750
**Age**	.001	.004	.384	.384		.006	-.003	.002	-1.033	.314		.070	.349
**Model**	N = 21, R^2 = .412, F = 4.0, p =.026*						N = 24, R^2 = .591, F = 9.6, p < .001*						
